# United Formula for the Friction Factor in the Turbulent Region of Pipe Flow

**DOI:** 10.1371/journal.pone.0154408

**Published:** 2016-05-02

**Authors:** Shuolin Li, Wenxin Huai

**Affiliations:** State Key Laboratory of Water Resources and Hydropower Engineering Science, Wuhan University, Wuhan, 430072, China; University at Buffalo, SUNY, UNITED STATES

## Abstract

Friction factor is an important element in both flow simulations and river engineering. In hydraulics, studies on the friction factor in turbulent regions have been based on the concept of three flow regimes, namely, the fully smooth regime, the fully rough regime, and the transitional regime, since the establishment of the Nikuradze’s chart. However, this study further demonstrates that combining the friction factor with Reynolds number yields a united formula that can scale the entire turbulent region. This formula is derived by investigating the correlation between friction in turbulent pipe flow and its influencing factors, i.e., Reynolds number and relative roughness. In the present study, the formulae of Blasius and Stricklerare modified to rearrange the implicit model of Tao. In addition, we derive a united explicit formula that can compute the friction factor in the entire turbulent regimes based on the asymptotic behavior of the improved Tao’s model. Compared with the reported formulae of Nikuradze, the present formula exhibits higher computational accuracy for the original pipe experiment data of Nikuradze.

## 1. Introduction

**Fig 1:**
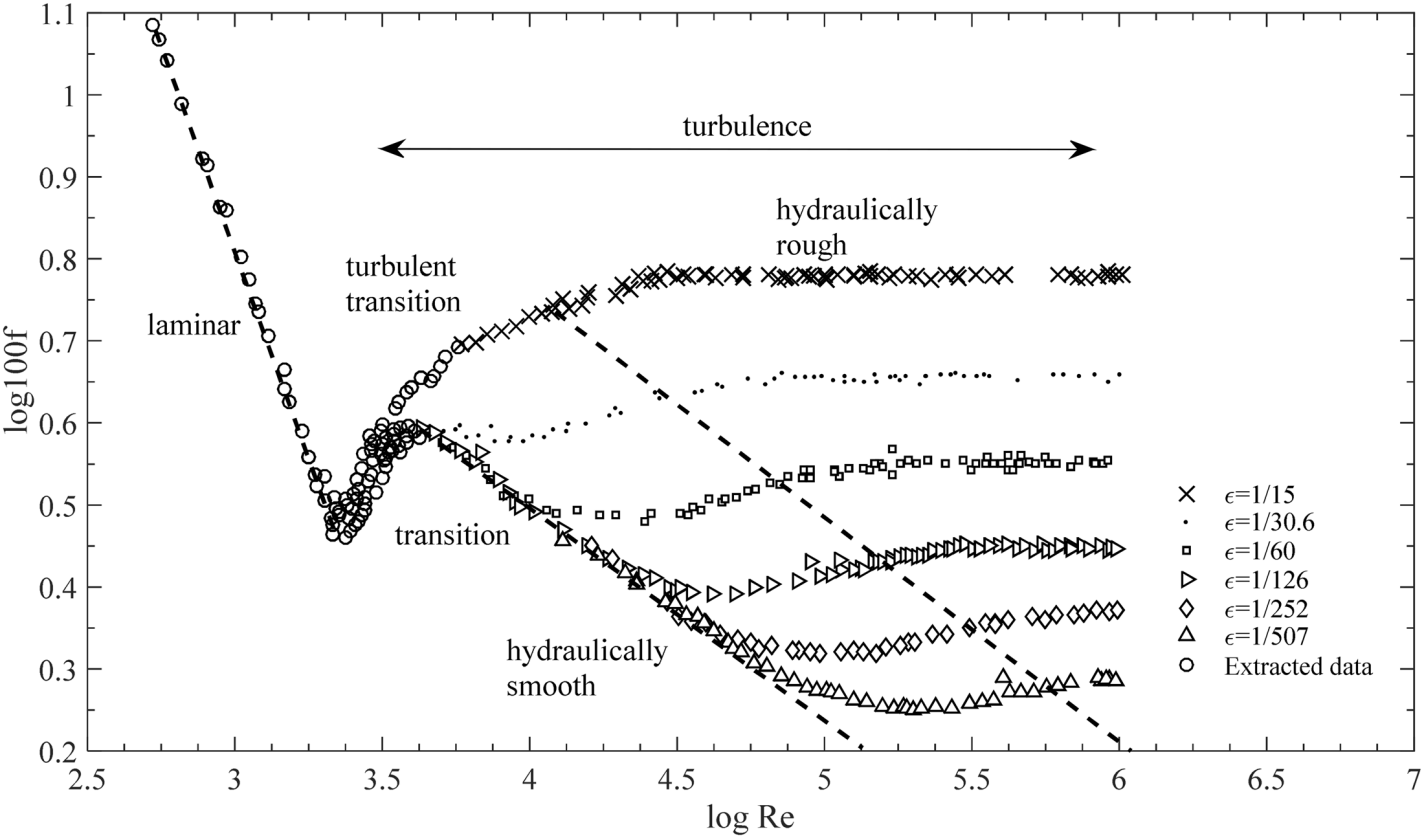
Friction factor of pipe flow in a rough pipe extracted from Nikuradze’s tabular and graphical presentation [2].

Fluid turbulence is one of the most intensively studied and most perplexing areas in classical physics [1]. This field comprises a host of properties that represent the most complicated aspects of our physical world: irregularity, diffusivity, rotational flow, and three-dimensionality. Previous researchers,such as Nikuradze [2], Blasius [3], and Strickler [4], have focused mainly on the interrelationship among several variables of turbulent flow, such as the Reynolds number *Re*, the roughness conditions *ε*, and the friction factor *f*. Nearly a century ago, Nikuradze conducted a series of experiments on pipe flow. He measured *f* against Re in various circular pipes that covered an extensive range of relative roughness *ε* values. Consequently, a comprehensive but nonlinear correlation among these three parameters was reported [2] and presented in a graph (Fig 1), called Nikuradze’s chart, which became a benchmark in the study of the friction factor in hydraulics.

In laminar pipe flow, resistance is caused solely by the viscosity shear stress[5]. The shear stress solved from the energy equation is presented as

$$\tau =\rho g\frac{r}{2}S$$ (1)

where *ρ* is the fluid density, *g*is the acceleration due to gravity, *r* is the radial coordinate measured from the center, and *S*is the hydraulic slope.

**Fig 2:**
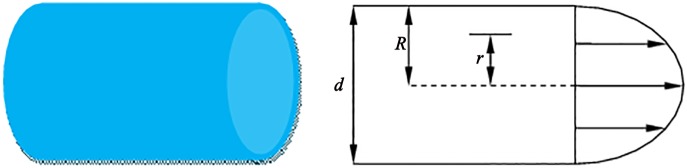
Diagram of the velocity distribution in a full-flow pipe [6].

Simultaneously, shear stress can also be computed from Newton’s law of inner friction [7] as follows (Fig 2):

$$\tau =\mu \frac{du}{dy}=-\mu \frac{du}{dr}$$ (2)

By substituting Eq (2) into Eq (1), we obtain *du* = − *ρgSrdr*/2*μ*. When this result is implemented across the entire section, we obtain mean velocity $U={\left(\pi R^{2}\right)}^{-1}\int_{0}^{R}u2\pi rdr=\rho gSd^{2}/32\mu$, which corresponds to the Darcy–Weisbach formula *f* = 2*gdS*/*U*^2^[8]. Hence, we determine *f* = 64/Re.

In the turbulence region, *f* passes through the hydraulically smooth, the transitional, and the hydraulically rough regions. In the hydraulically smooth region, the relationship between *f* and Re is *f ~* Re^−1/4^ according to Blasius [3]. When *f* Re = 64 in the laminar region, we also maintain the form of *f* Re; thus, *f* Re ~ Re^3/4^ is written for a fully smooth regime. In the hydraulically rough region, the relationship between *f* and *ε* is *f* ~ *ε*^1/3^, as suggested by Strickler [4]. Similarly, we obtain *f* Re ~ Re*ε*^1/3^. Tao [9] proposed an implicit function *G*(*x*) based on these two form-changed formulae to rescale Fig 1 as follows:

$$f\text{Re}=G({\text{Re}}^{3/4}+C_{s}{\text{Re}}^{\varsigma }\;\varepsilon^{\varsigma /3})$$ (3)

where *ξ* = 2 and *C*_*s*_ = 3×10^−5^ are adjustable parameters computed by Tao based on the degree of discreteness [9] of the data. *G(x)* is an implicit function with certain characteristics that conform to the boundary conditions. This function is discussed in the following section.

## 2. Interpolation Method

### 2.1 Model Modification

Recently, Gioia *et al*. [10] modified Strickler’s formula and revised the relationship into *f* ~ *ε*^*α*^, where *α* = 1/3 + *η*/2, and *η* = 0.02 was calculated by Mehrafarin and Pourtolamiilarly in a phenomenon argument [11] by modifying the finding of Goldenfeld [12]. Thus, Strickler’s formula can be modifiedinto *f* Re ~ *ε*^*α*^Re. When the revision proposed by Gioia *et al*. [10] is considered, Tao’s formula [9] can be revised into

$$f\text{Re}=G({\text{Re}}^{3/4}+C_{s}{\text{Re}}^{\varsigma }\varepsilon^{\varsigma \alpha })$$ (4)

We observed the limited condition of Eq (4) and found that when Re was relatively small, as hinted by Tao [9], *C*_*s*_ was used to ensure *C*_*s*_ Re^*ς*^ *ε*^*ςα*^ → 0; hence, Eq (4) became *f* Re = *G*(Re^3/4^). Consequently, the requirements *f* Re ~ Re^3/4^ for Blasius’ formula and *f* Re ~ (Re)^0^ for laminar flow can be fulfilled, which is consistent with the laminar regime. When Re is extremely large, Eq (4) can be written as *f* Re ~ *G*[Re^*ς*^(Re^3/4−*ς*^ + *C*_*s*_*ε*^*ςα*^)]. In this case, *ς* > 3/4 is required to guarantee Re^3/4−*ς*^ → 0 or *f* Re ~ *G*[Re^*ς*^ *C*_*s*_*ε*^*ςα*^)]; thus, to maintain Eq (4)coordination with the revised Strickler’s formula, only *G*(Re^*ς*^ *C*_*s*_*ε*^*ςα*^)~(Re^*ς*^ *C*_*s*_*ε*^*ςα*^)^1/*ς*^is required.

**Fig 3:**
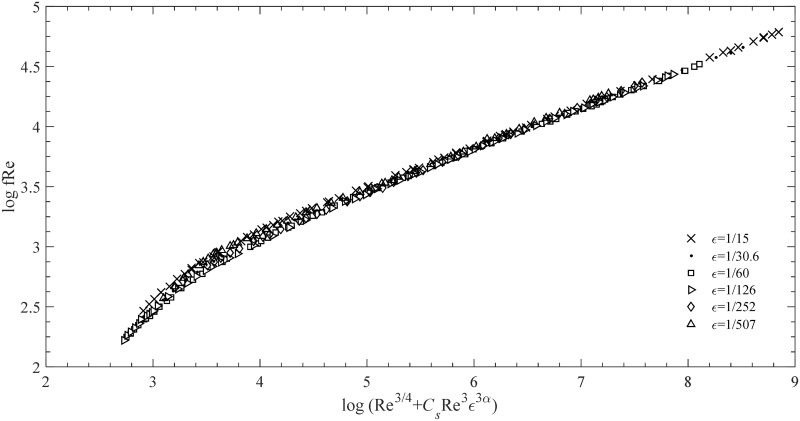
Data of Nikuradze’s experiment rescaled using Eq (4).

Now, we apply Eq (4) to the turbulent regime, i.e., Eq (4), along with Nikuradze’s turbulence data, as shown in Fig 3. In this regime, *C*_*s*_ = 1×10^−8^ and *ξ* = 3 are obtained based on the least squares procedure.

### 2.2 Explicit Formula

In Fig 3, the experimental points generally converge onto a monotonous curve that simplifies Nikuradze’s chart. This curve provides further insight into the dependence of *f*on Re and ε. Moreover, if this curve is extended at both ends, then its two sides asymptote to two straight lines. That is, when the limit Re is regarded as zero, the parameter *C*_*s*_ Re^3^ *ε*^3*α*^ tends to be zero relative to Re^3/4^. In this case, we have $\underset{\text{Re}\to 0}{\text{lim}}C_{S}{\text{Re}}^{3}\varepsilon^{3\alpha }/{\text{Re}}^{3/4}=C_{S}\varepsilon^{3\alpha }\;\underset{\text{Re}\to 0}{\text{lim}}{\text{Re}}^{9/4}=0$. Thus, Eq (4) is reduced to *f* Re = *G*(Re^3/4^). To conform to Blasius’ formula *f ~* Re^−1/4^ [3], or equivalently, *f* Re *~* Re^3/4^, *G*(*x*) should be a linear function. That is, Eq (4) should asymptote into a straight line with a gradient of 1 in a log–log plot. The expression fitted to the experiment data can be written as

$${\text{log}}_{10}(f\text{Re})=K_{1}{\text{log}}_{10}(x)+C_{1}$$ (5)

where *K*_1_ = 1,*C*_1_ = −0.5098,and *x* = Re^3/4^ *+C*_*s*_ Re^3^ *ε*^3*α*^.

We now relate this to Eq (4) by obtaining *G*(Re^3/4^+*C*_*s*_Re^3^*ε*^3*α*^) ~ *G*[Re^3^(Re^9/4^+*C*_*s*_*ε*^3*α*^)]. For large Reynolds numbers, an equation similar to Eq (3) must satisfy the revised Strickler’s formula [4], namely, *f* ~ *ε*^*α*^,or equivalently, *f* Re *~ ε*^*α*^ Re. Thus, Eq (4) should take the form of *G*(Re^3/4^ *+C*_*s*_ Re^3^ *ε*^3*α*^)~[Re^3^(Re^−9/4^ *+C*_*s*_*ε*^3*α*^)]^1/3^ (in this case, Re^−9/4^ can be regarded as zero). Therefore, we derive an explicit expression for the linear asymptote at a large Re (this expression can also be adopted when turbulence is fully developed):

$${\text{log}}_{10}(f\text{Re})=K_{2}{\text{log}}_{10}(x)+C_{2}$$ (6)

where *K*_*1*_ = 1/3and *C*_2_ = −1.825

Given these two tending character of the curves in Fig 1, we combine Eq (5) and Eq (6) to establish

$${\text{log}}_{10}\left(f\text{Re}\right)=K_{1}{\text{log}}_{10}x+\frac{K_{2}-K_{1}}{\beta }{\text{log}}_{10}\left[1+{\left(\frac{x}{x_{0}}\right)}^{\beta }\right]+C_{1}$$ (7)

where log_10_*x*_0_ = (*C*_1_−*C*_2_)/(*K*_2_−*K*_1_), and *β* is the transitional shape parameter first used by Guo [13]. The turbulence region lies between two extended lines; hence, Eq (7) is accessible in the turbulence region. The shape parameter can be determined by using the collocation method suggested by Griffiths and Smith [13]. In particular, for *x*<<*x*_0_, log_10_[1+(*x*/*x*_0_)^*β*^]→0, then Eq (7) is transformed into Eq (5); for *x*>>*x*_0_, log_10_[1+(*x*/*x*_0_)^*β*^]→*β*(log_10_ *x*−log_10_ *x*_0_), then Eq (7) is transformed into Eq (6).

**Fig 4:**
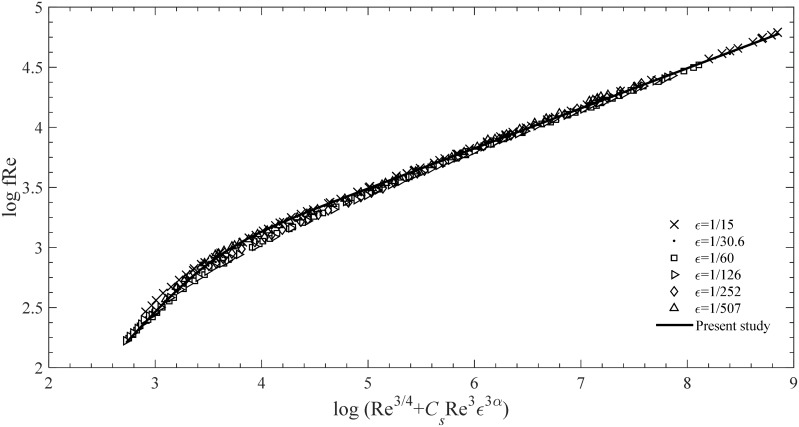
Comparison of the curves obtained from Tao’s model for various ε values with Eq (6).

After validating Eq (7) with specific data [14], we obtain an integrated expression for the friction factor that covers an extensive range of turbulence region as follows:

$$f=\frac{x}{3.24\text{Re}{\left[1+{\left(x/3178\right)}^{8/5}\right]}^{5/12}}$$ (8)

which is plotted in Fig 4, where *β* = 8/5.

### 2.3 Comparison with Nikuradze’s Formulae

In deriving Eq (8), *f* Re (the product of the friction factor and the Reynolds number) can be regarded as a single parameter to establish an improved mathematical law. Hence, the relationship among *f*, Re, and *ε* becomes a relationship among *f* Re, Re^3/4^, and Re^3^ *ε*^3*α*^; such a relationship provides an easier representation of the data to be studied (compare Fig 1 with Fig 4). Therefore, when comparing the results of the present study with those from the original data or the previous formulae, we adopt *f* Re to replace the single *f*, thereby verifying the accuracy of our analysis in a clear and convenient manner.

**Fig 5:**
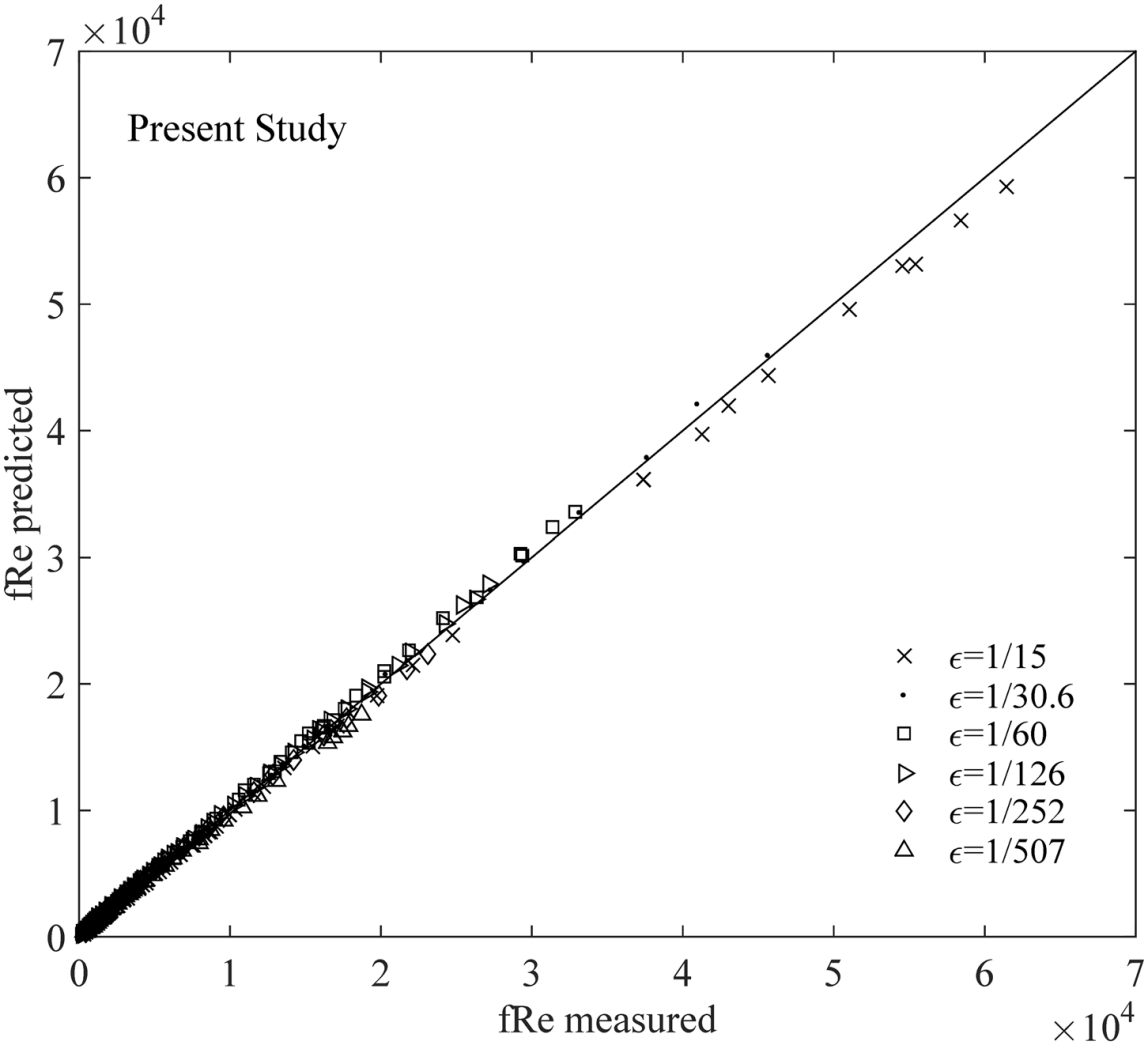
Comparison between the results of the present study and the experimental data for the entire turbulence regime.

First, the values of *f* Re that are calculated using Eq (8) are compared with those obtained from the experimental data of Nikuradze for the entire turbulence region. The result presented in Fig 5 and Table 1 shows that Eq (8) exhibits a strong linearity for the entire turbulent regime.

**Table 1:** Prediction Errors for Different Formulae.

Average relative errors of the friction factor (%)		
Investigator	Nikuradze	Present theory
Equation	(9),(10)	(8)
Entire turbulence region	None	5.4
Smooth zone	30.8	3.2
Rough zone	20.1	4.3

**Fig 6:**
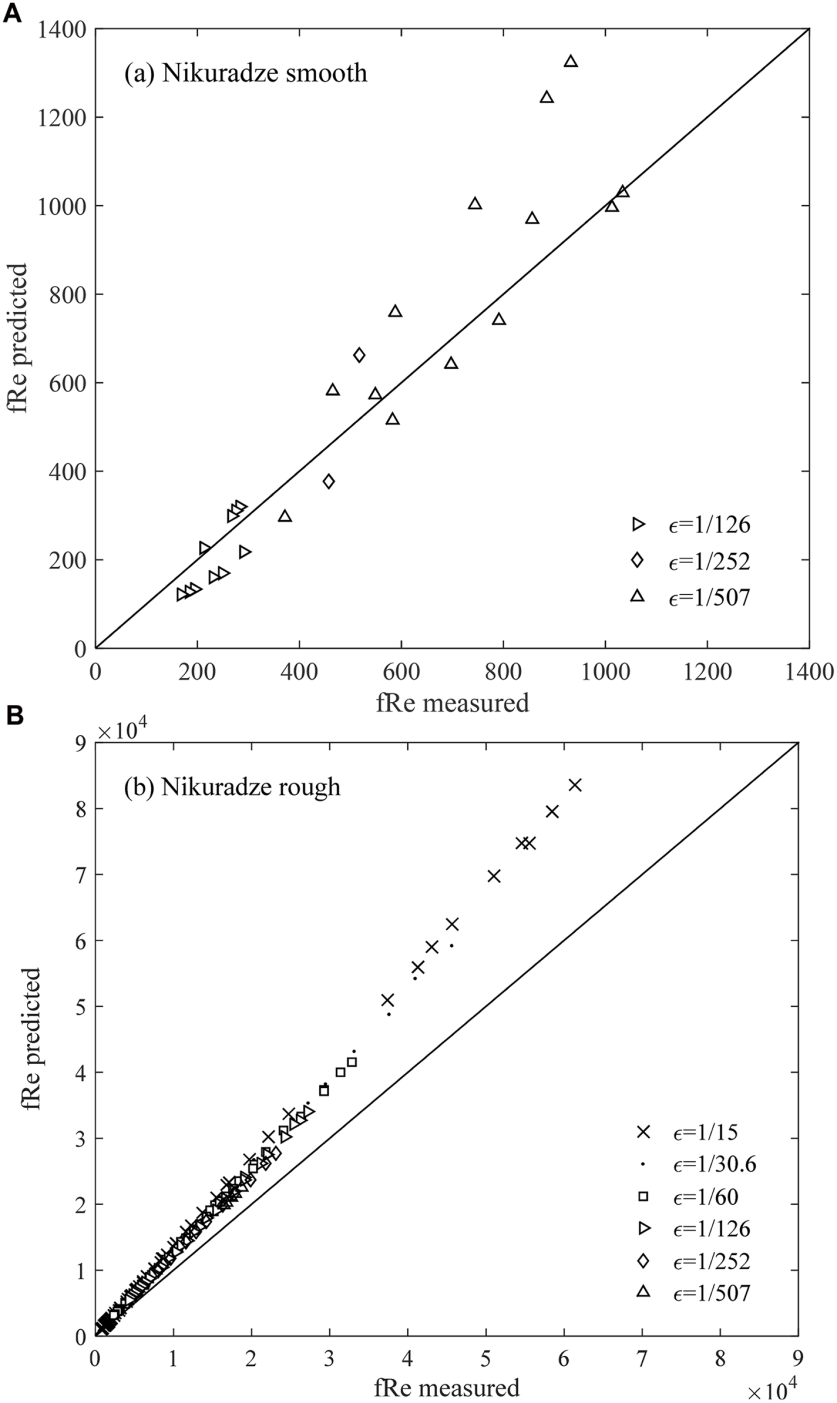
Comparison between Nikuradze’s formulae and the experimental data for the smooth and rough turbulence zones.

Moreover, Nikuradze’s formulae for the smooth zone and the rough zone are compared with the data from his experiments (Fig 6). Nikuradze’s formulae are [6],

$$\frac{1}{\sqrt{f}}=2\text{lg}\left(\text{Re}\sqrt{f}\right)-0.8\;$$ (9)

for $\sqrt{f/8}\text{Re}\varepsilon <5$, i.e., in the hydraulically smooth turbulence zone, and

$$f=\frac{1}{{\left[2\text{lg}\left(3.71/\varepsilon \right)\right]}^{2}}$$ (10)

for $\sqrt{f/8}\text{Re}\varepsilon >70$, i.e., in the hydraulically rough turbulence zone.

**Fig 7:**
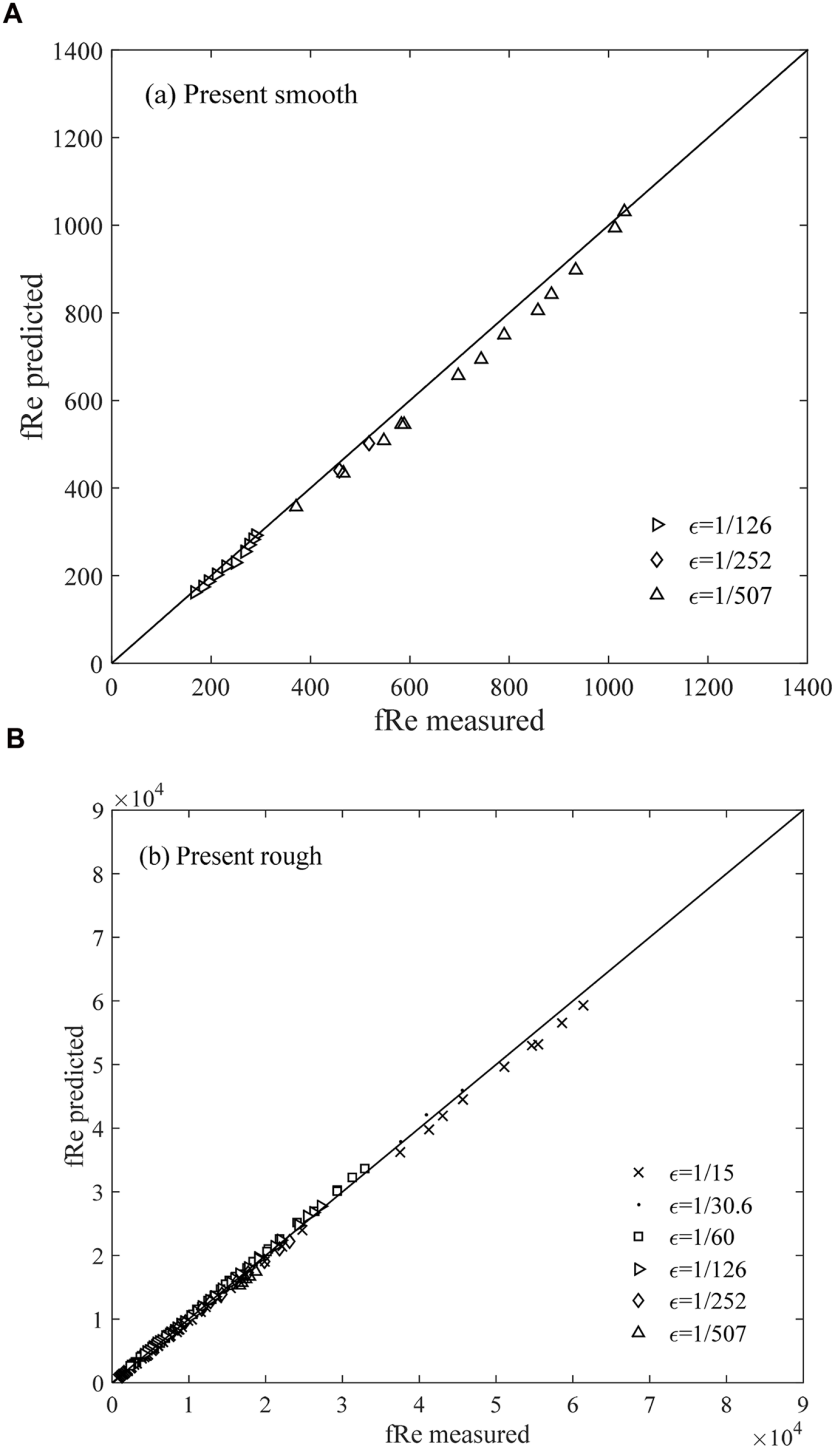
Comparison between the present study and the experimental data for the smooth and rough turbulence zones.

Finally, the values of *f* Re predicted using Eq (8) are also validated against the experimental data of Nikuradze for both smooth and rough zones (Fig 7).

Meanwhile, the relative errors computed as |*measured*−*predicted*|/|*measured*| in the aforementioned figures (Figs 5–7) are listed in Table 1. This table shows that the *f* value from Eq (8), which has an error of 5.4%, is applicable in calculating or predicting the friction factor for different turbulent pipe flows. We suggest that Eq (8) is a useful and reliable method for hydraulic research and applications. The result shows that the relative error obtained from Nikuradze’s formula (9) for the hydraulically smooth turbulence region is 30.8%, which is nearly 10 times higher than that obtained from Eq (8). The relative error of Nikuradze’s formula (10) is 20.1%, which is thrice higher than that obtained from Eq (8). Therefore, the prediction of the present study for the friction factor *f* (or *f* Re) is significantly more reliable than that of Nikuradze’s formulae for the two boundary zones. Moreover, unlike Eq (8), Nikuradze did not provide a formula for the transition zone. A single formula that covers all the three zones is clearly more convenient for calculations. Furthermore, Nikuradze’s formula (9) is an implicit expression for *f*, whereas Eq (8) is explicit.

## 3. Discussion

In the past, the calculation and analysis of the friction factor *f* has been a consistent concern among hydraulic researchers because of the significance of this factor in understanding pipe flow and sediment transport. Accordingly, several formulae (Colebrook [15]; Brownlie [16]; Cheng and Chiew [17]; Ligrani and Moffat [18]; Yalin and daSilva [19]) have been proposed in the literature to estimate the friction factor; however, they must be computed separatelyunder laminar, fully smooth, and rough turbulent flow conditions. Compared with these formulae, the proposed formula can scale the entire turbulent regimes, and thus, is definitely more practical to use. To the best of our knowledge, no single formula that canexplicitly calculate the friction factor in various flow regimeshas yet been established, except for the combination approach of Cheng [20]. In his study, the friction factor was assumed to have the function form of $f=f_{L}^{\partial }f_{T}^{1-\partial }$, where *f*_*L*_ is a friction factor for laminar flow, *f*_*T*_ is that for turbulent flow, and ∂ is the weighing factor. However, the present formula is based on the combination of a new parameter, i.e., *f* Re, in which we do not have to consider the flow regimes. Therefore,the proposed formula is entirely different from Cheng’s formulae.

Motivated by the idea of deriving a single monotonic function, we developed an explicit expression for the friction factor of pipe flow that covered the entire Re range by interpolating the two asymptotic expressions into a single monotonic function through the rescaling the experimental data of Nikuradze. The comparisons between the curves of the data suggest that the predictions obtained using our formula are accurate and reliable, including those that correspond to the transition zone of the original Nikuradze chart. In this study, we have noted and verified that parameter *f* Re should be regarded as a relevant parameter by checking it against the boundary conditions for Re and *ε*. A revised rescaled function (Tao [9]) is then possible. This method is proven to be highly helpful in explicitly uncovering the dependence of the friction factor. In hydraulics, the results provided by Nikuradse’s experiments have served as the basis of research on friction resistance. The concepts of a hydraulically smooth zone, a hydraulic transitional zone, and a hydraulically rough zone have been used for nearly a century to study the friction factor given the lack of knowledge on the united relationship among the three zones. Thus, this study is the first to unite these three zones and to provide a united formula that can scale the entire turbulence regime. The convenience brought by uniting the empirical equations does not only considerably aid in the computation of hydraulic parameters, such as frictional head loss, but also further enhances the understanding of flow resistance.

## Supporting Information

S1 File Nikuradse’s original paper. (PDF)

S2 File Nikuradse's original experimental data. (XLS)
