# Supplementary material for: United Formula for the Friction Factor in the Turbulent Region of Pipe Flow
Source: PLoS One. 2016 May 2;11(5):e0154408. doi: 10.1371/journal.pone.0154408 (PMC4852912; doi:10.1371/journal.pone.0154408)
Supplement: S1 File — (PDF) [file pone.0154408.s001.pdf]

NACA TM 1292

NATIONAL ADVISORY COMMITTEE FOR AERONAUTICS

TECHNICAL MEMORANDUM 1292

LAWS OF FLOW IN ROUGH PIPES

By J. Nikuradse

Translation of "Strömungsgesetze in rauhen Rohren." VDI-Forschungsheft 361. Beilage zu "Forschung auf dem Gebiete des Ingenieurwesens" Ausgabe B Band 4, July/August 1933.

Washington
November 1950

NATIONAL ADVISORY COMMITTEE FOR AERONAUTICS

TECHNICAL MEMORANDUM 1292

LAWS OF FLOW IN ROUGH PIPES*

By J. Nikuradse

INTRODUCTION

Numerous recent investigations (references 1, 2, 3, 4, and 5) have greatly increased our knowledge of turbulent flow in smooth tubes, channels, and along plates so that there are now available satisfactory data on velocity distribution, on the laws controlling resistance, on impact, and on mixing length. The data cover the turbulent behavior of these flow problems. The logical development would now indicate a study of the laws governing turbulent flow of fluids in rough tubes, channels, and along rough plane surfaces. A study of these problems, because of their frequent occurrence in practice, is more important than the study of flow along smooth surfaces and is also of great interest as an extension of our physical knowledge of turbulent flow.

Turbulent flow of water in rough tubes has been studied during the last century by many investigators of whom the most outstanding will be briefly mentioned here. H. Darcy (reference 6) made comprehensive and very careful tests on 21 pipes of cast iron, lead, wrought iron, asphalt-covered cast iron, and glass. With the exception of the glass all pipes were 100 meters long and 1.2 to 50 centimeters in diameter. He noted that the discharge was dependent upon the type of surface as well as upon the diameter of the pipe and the slope. If his results are expressed in the present notation and the resistance factor λ is considered dependent upon the Reynolds number Re, then it is found that according to his measurements λ, for a given relative roughness $\frac{k}{r}$, varies only slightly with the Reynolds number (k is the average depth of roughness and r is the radius of the pipe; Reynolds number $Re = \bar{u}\frac{d}{\nu}$ in which $\bar{u}$ is the average velocity, d is the pipe diameter, and ν is the kinematic viscosity). The friction factor decreases with an increasing Reynolds number and the rate of decrease becomes slower for greater relative roughness. For certain roughnesses his data indicate that the friction factor λ is independent of the Reynolds number.

---

*"Strömungsgesetze in rauhen Rohren." VDI-Forschungsheft 361. Beilage zu "Forschung auf dem Gebiete des Ingenieurwesens" Ausgabe B Band 4, July/August 1933.

For a constant Reynolds number, λ increases markedly for an increasing relative roughness. H. Bazin (reference 7), a follower of Darcy, carried on the work and derived from his own and Darcy's test data an empirical formula in which the discharge is dependent upon the slope and diameter of the pipe. This formula was used in practice until recent times.

R. v. Mises (reference 8) in 1914 did a very valuable piece of work, treating all of the then-known test results from the viewpoint of similarity. He obtained, chiefly from the observations of Darcy and Bazin with circular pipes, the following formula for the friction factor λ in terms of the Reynolds number and the relative roughness:

$$\lambda = 0.0024 + \sqrt{\frac{k}{r}} + \frac{0.3}{\sqrt{Re}}$$

This formula for values of Reynolds numbers near the critical, that is, for small values, assumes the following form:

$$\lambda = \left(0.0024 + \sqrt{\frac{k}{r}}\right)\left(1 - \frac{1000}{Re}\right) + \frac{0.3}{\sqrt{Re}}\sqrt{1 - \frac{1000}{Re}} + \frac{8}{Re}$$

The term "relative roughness" for the ratio $\frac{k}{r}$ in which k is the absolute roughness was first used by v. Mises. Proof of similarity for flow through rough pipes was furnished in 1911 by T. E. Stanton (reference 9). He studied pipes of two diameters into whose inner surfaces two intersecting threads had been cut. In order to obtain geometrically similar depths of roughness he varied the pitch and depth of the threads in direct proportion to the diameter of the pipe. He compared for the same pipe the largest and smallest Reynolds number obtainable with his apparatus and then the velocity distributions for various pipe diameters. Perfect agreement in the dimensionless velocity profiles was found for the first case, but a small discrepancy appeared in the immediate vicinity of the walls for the second case. Stanton thereby proved the similarity of flow through rough tubes.

More recently L. Schiller (reference 10) made further observations regarding the variation of the friction factor λ with the Reynolds number and with the type of surface. His tests were made with drawn brass pipes. He obtained rough surfaces in the same manner as Stanton by using threads of various depths and inclinations on the inside of the test pipes. The pipe diameters ranged from 8 to 21 millimeters. His observations indicate that the critical Reynolds number is independent of the type of wall surface. He further determined that for greatly roughened surfaces the quadratic law of friction is effective as soon

as turbulence sets in. In the case of less severely roughened surfaces he observed a slow increase of the friction factor with the Reynolds number. Schiller was not able to determine whether this increase goes over into the quadratic law of friction for high Reynolds numbers, since the Göttingen test apparatus at that time was limited to about $Re = 10^5$. His results also indicate that for a fixed value of Reynolds number the friction factor λ increases with an increasing roughness.

L. Hopf (reference 11) made some tests at about the same time as Schiller to determine the function $\lambda = f\left(Re\ \frac{k}{r}\right)$. He performed systematic experiments on rectangular channels of various depths with different roughnesses (wire mesh, zinc plates having saw-toothed type surfaces, and two types of corrugated plate). A rectangular section was selected in order to determine the effect of the hydraulic radius (hydraulic radius r' = area of section divided by wetted perimeter) on the variation in depth of section for a constant type of wall surface. At Hopf's suggestion these tests were extended by K. Fromm (reference 12). On the basis of his own and Fromm's tests and of the other available test data, Hopf concluded that there are two fundamental types of roughness involved in turbulent flow in rough pipes. These two types, which he terms surface roughness and surface corrugation, follow different laws of similarity. A surface roughness, according to Hopf, is characterized by the fact that the loss of head is independent of the Reynolds number and dependent only upon the type of wall surface in accordance with the quadratic law of friction. He considers surface corrugation to exist when the friction factor as well as the Reynolds number depends upon the type of wall surface in such a manner that, if plotted logarithmically, the curves for λ as a function of the Reynolds number for various wall surfaces lie parallel to a smooth curve. If a is the average depth of roughness and b is the average distance between two projections from the surface, then surface corrugation exists for small values of $\frac{a}{b}$ and surface roughness exists for large values of $\frac{a}{b}$.

A summary of the tests of Hopf, Fromm, Darcy, Bazin and others is given in figures 1 and 2, the first illustrating surface roughness and the second surface corrugation. Hopf derived for the friction factor λ within the range of surface roughness the following empirical formula:

$$\lambda = 4 \times 10^{-2}\left(\frac{k}{r'}\right)^{0.314}$$

in which r' is the hydraulic radius of the channel $\left(r' = \frac{2F}{U};\ F = \text{area of cross-section};\ U = \text{wetted perimeter}\right)$. This formula applies to iron pipes, cement, checkered plates and wire mesh. In the case of surface

corrugation he gives the formula

$$\lambda = \lambda_o \xi$$

in which λ_o is the friction factor for a smooth surface and ξ is a proportionality factor which has a value between 1.5 and 2 for wooden pipes and between 1.2 and 1.5 for asphalted iron pipes.

The variation of the velocity distribution with the type of wall surface is also important, as well as the law of resistance. Observations on this problem were made by Darcy, Bazin, and Stanton (reference 9). The necessary data, however, on temperature of the fluid, type of wall surface, and loss of head are lacking. In more recent times such observations have been made by Fritsch (reference 13) at the suggestion of Von Kármán, using the same type of apparatus as that of Hopf and Fromm. The channel had a length of 200 centimeters, width of 15 centimeters and depth varying from 1.0 to 3.5 centimeters. A two-dimensional condition of flow existed, therefore, along the short axis of symmetry. He investigated the velocity distribution for the following types of wall surface:

1. smooth
2. corrugated (wavy)
3. rough
   I. (floors, glass plates with light corrugations)
4. rough
   II. (ribbed glass)
5. toothed (termed saw-toothed by Fromm)

Fritsch found that for the same depth of channel the velocity distribution (except for a layer adjacent to the walls) is congruent for all of these types of surfaces if the loss of head is the same.

Tests in a channel with extremely coarse roughness were made by Treer, (references 14 and 15) in which he observed the resistance as well as the velocity distribution. From these tests and from those of other investigators, he found that the velocity distribution depends only upon the shearing stress, whether this is due to variation in roughness or in the Reynolds number.

The numerous and in part very painstaking tests which are available at the present time cover many types of roughness, but all lie within a

very small range of Reynolds number. The purpose of the present investigation is to study the effect of coarse and fine roughnesses for all Reynolds numbers and to determine the laws which are indicated. It was, therefore, necessary to consider a definite relative roughness $\frac{r}{k}$ for a wide range of Reynolds number and to determine whether for this constant $\frac{r}{k}$, that is, for geometrical similarity, the value $\lambda = f(Re)$ is the same curve for pipes of different diameter. There was also the question whether for the same $\frac{r}{k}$ the velocity distributions are similar and vary with the Reynolds number, and whether for a varying $\frac{r}{k}$ the velocity distributions are similar as stated by V. Kármán.

I wish here to express my sincere gratitude to my immediate superior, Professor Dr. L. Prandtl, who has at all times aided me by his valuable advice.

I. EXPERIMENT

1. Description of Test Apparatus

The apparatus shown in figure 3 was used in making the tests. The same apparatus was employed in the investigation of velocities for turbulent flow in smooth pipes. The detailed description of the apparatus and measuring devices has been presented in Forschungsheft 356 of the VDI. Only a brief review will be given here. Water was pumped by means of a centrifugal pump kp, driven by an electric motor em, from the supply canal vk, into the water tank wk, then through the test pipe vr and into the supply canal vk. This arrangement was employed in the investigation with medium and large values of Reynolds number. An overflow was used in obtaining observations for small values of Reynolds number. The water flowed through the supply line zl, into the open water tank wk, and a vertical pipe str, connected with the tank, conducted the overflowing water over the trap and down through the overflow pipe fr. The flow in the test pipe could be throttled to any desired degree. A constant high pressure in the water tank wk was required in order to attain the highest values of Reynolds number. Observations were made on:

1. loss of head

2. velocity distribution in the stream immediately after leaving the test pipe

3. discharge quantity

4. temperature of the water

Three hooked tubes with lateral apertures were used to measure the loss of head. These tubes are described in detail in section I,3. The velocity distribution was determined by means of a pitot tube with 0.2 millimeter inside diameter, mounted in the velocity-measuring device gm, and adjustable both horizontally and vertically. The discharge for Reynolds numbers up to 3×10^5 was measured in a tank mb on the basis of depth and time. Larger discharges were computed by integrating the velocity distribution curve. Temperature readings were taken at the outlet of the velocity-measuring device gm. The test pipes were drawn brass pipes of circular section whose dimensions are given in table 1. The diameters of the pipe were determined from the weight of the water which could be contained in the pipe with closed ends and from the length of the pipe.

2. Fabrication and Determination of Roughness

Similitude requires that if mechanically similar flow is to take place in two pipes they must have a geometrically similar form and must have similar wall surfaces. The first requirement is met by the use of a circular section. The second requirement is satisfied by maintaining a constant ratio of the pipe radius r to the depth k of projections. It was essential, therefore, that the materials producing the roughness should be similar. Professor D. Thoma's precedent of using sand for this purpose was adopted.

Grains of uniform size are required to produce uniform roughness throughout the pipe. Ordinary building sand was sifted. In order to obtain an average grain size of 0.8 millimeter diameter, for example, sieves were employed having openings of 0.82- and 0.78-millimeter diameter. A Zeiss thickness gage was used to obtain the actual average grain size by taking actual measurements of the diameter of several hundred grains. These sand grains were spread on a flat plate. The diameters of the individual grains were then measured with the Zeiss thickness gage (having an accuracy of 0.001 mm) by sliding the plate. For the case cited the arithmetical average was found to be 0.8 millimeter.

A micro-photograph of uniform size (0.8-mm diameter) grains as reproduced in figure 4 furnishes some information regarding grain form. Preliminary tests had indicated the manner in which the pipes could be roughened with sand. The pipe placed in a vertical position and with the lower end closed was filled with a very thin Japanese lacquer and then emptied. After about 30 minutes, which is a period sufficient for the drying of the lacquer on the pipe surface to the "tacky" state,

the pipe was filled with sand of a certain size. The sand was then allowed to flow out at the bottom. The preliminary tests showed that the drying which now follows is of great importance for durability. A drying period of two to three weeks is required, depending upon the amount of moisture in the air. A uniform draft in the pipe, due to an electric bulb placed at the lower end, helped to obtain even drying. After this drying, the pipe was refilled with lacquer and again emptied, in order to obtain a better adherence of the grains. There followed another drying period of three to four weeks. At each end of the pipe, a length of about 10 centimeters was cut off in order to prevent any possible decrease in the end sections. After the treatment just described the pipes were ready to be measured.

One of the conditions cited above indicates that different grain sizes must be used for pipes of different diameter if the ratio $\frac{r}{k}$, which is the gage for similarity of wall surface, is to remain constant. Geometrical similarity of the wall surface requires that the form of the individual grains shall be unchanged and also that the projection of the roughening, which has hydrodynamical effects, shall remain constant. Figure 4 shows that voids exist between the grains. The hydrodynamically effective amount of projection k is equal to the grain size. In order to determine whether the previously observed diameter of grains is actually effective, a flat plate was coated with thin Japanese lacquer (the necessary degree of thinness was determined by preliminary tests) and roughened in accordance with the described procedure. The projection of the grains above the surface was measured in the manner already described and it was found that, for a definite degree of thinness of the lacquer, this average projection agreed with the original measurements of the grains.

3. Measurement of Static Pressure Gradient

Measurement of static pressure gradient during flow in smooth pipes is usually made by piezometer holes in the walls of the pipe. Marked errors result, however, if loss of head in rough pipes is determined in this same manner. These are due to the fact that the vortices which result from flow around the projections produce pressure or suction, depending on the position of the aperture. For this reason the hooked tube was adopted for observing the static pressure gradient. This tube had a rectangular bend as shown in figure 5 and was mounted in the test pipe so that the free leg was parallel to the direction of flow. Lateral openings only were bored in this free leg. The outside diameter d of the tube was 2 millimeters. Other features of the tube are in agreement with the specifications (reference 16) set up for the Prandtl pitot static tube (Staurohr). The free leg was placed at a distance from the wall equal to 1/2 the radius of the test pipe. The

connecting leg was bent at an angle of about 60° in the plane of the free leg in order that the position of the free leg might always be indicated. The bent tube was fastened in the test pipe by means of a stuffing box.

Variation of the pressure readings in a hooked tube with variations in the position of the tube relative to the direction of flow is shown in figure 6[1]. This figure indicates that correct readings are obtained only if the direction of the free leg deviates not more than 7.5° from the direction of flow. The introduction of the hooked tube into the test pipe results in an increase of pressure drop due to the resistance to the tube. The resistance of the two hooked tubes used in measuring must be deducted from the observed pressure drop $p_1 - p_2$. The resistance of the tube must therefore be known. This value was found by measuring the pressure drop h in a smooth pipe in terms of the discharge at a constant temperature, first by using wall piezometer orifices and then by measuring the pressure drop $h + a$ in terms of the discharge at the same temperature by means of a hooked tube. The increment a for equal discharges is the resistance of the hooked tubes. The correction curve for this resistance is given in figure 7.

It should be noted that changes in direction of the tube result both in an error in the pressure reading and in an increase in the resistance due to the tube. If the corrected pressure drop $p_1 - p_2$ is divided by the observation length l, (distance between the holes in the side of the hooked tubes), there is obtained the static pressure gradient,

$$\frac{dp}{dx} = \frac{p_1 - p_2}{l}$$

4. Preliminary Tests

A mixture of sieved sand and white lacquer in a definite proportion was used to fill a pipe closed at the bottom, in the manner of Professor D. Thoma (reference 17). The mixture was then allowed to flow out at the bottom. After a drying period of about two to three weeks, preliminary tests answered the question whether the hydrodynamically effective projection of the roughening remained constant. The pressure drop was measured at hourly intervals for a given Reynolds number for which the

[1]This figure is taken from the work of H. Kumbruch, cited herein as reference 16.

average velocity $\bar{u}$ was about 20 meters per second. It was observed that within a few days the pressure slope developed a pronounced increase. A marked washing off of the lacquer was indicated at the same time by deposits on the bottom of the supply channel. Another objectionable feature was the partial washing out of the sand. The increase in the pressure gradient is accounted for by the increase in projection of roughness due to the washing off of the lacquer. Therefore, the method of fastening the sand had to be changed in order to insure the required condition of the surface during the test procedure. The projection k of the roughness had to remain constant during the tests and the distribution of the sand grains on the wall surfaces had to remain unchanged.

Adhesion between sand grains was prevented by using a very thin lacquer. This lacquer formed a direct coating on the wall and also a covering on the grains no thicker than the penetration of these grains into the lacquer coating of the wall. The original form and size of the grains remained unchanged. A determining factor in this problem was the degree of thickness of the lacquer which was varied by the addition of turpentine until the original grain size remained unchanged. Tests made with pipes without lacquer recoating showed that the sand would wash out. The recoating with lacquer was, therefore, adopted. If only a short period of drying was used for both coats, the lacquer was washed off. If the first drying was short and the second long, then all of the lacquer was also washed off. If the first drying period were long and the second short, there would also be some loss of sand. A constant condition of roughness could be obtained only when each lacquer coating was dried from three to four weeks. The accuracy of observations made with the hooked tube was checked by connecting the tube through a manometer to a wall piezometer orifice at the same section of the pipe. Both connections should show the same pressure in a smooth pipe, that is, the manometer reading must be zero. Hooked tubes checked in this manner were used for taking principal observations.

Finally, a determination of the approach length $\frac{x}{d}$ was made. Velocity distributions were observed for the largest relative roughness ratio $\frac{k}{r} = \frac{1}{15}$. The velocity at various distances y from the surface was determined for Reynolds numbers of $Re = 20 \times 10^3$, 70×10^3, and 150×10^3 at various distances from the entrance $\frac{x}{d}$. This was effected by cutting off portions of the test pipe. Tests show that changes in the approach length have small effect on the Reynolds number. The approach length is somewhat shorter than that for smooth pipes, $\frac{x}{d} \approx 40$ (fig. 8). The approach length $\frac{x}{d} = 50$ was used as for smooth pipes.

II. EVALUATION OF TEST RESULTS

1. Law of Resistance

The resistance factor λ for flow in the pipes is expressed by the formula:

$$\lambda = \frac{dp}{dx}\frac{d}{\bar{q}} \tag{1}$$

in which $\frac{dp}{dx}$ is the pressure drop per unit of length, d is the diameter, and $\bar{q} = \rho \frac{\bar{u}^2}{2}$, the dynamic pressure of the average flow velocity $\bar{u}$ and ρ is the density. An extensive test program with a range of $Re = 600$ to $Re = 10^6$ for the Reynolds number was carried out, and the relationship of the resistance factor to the Reynolds number was studied for pipes of various roughnesses. Six different degrees of relative roughness were used, with the relative roughness $\frac{k}{r}$ determined by the ratio of the average projection k to the radius r of the pipe.

In evaluating the test data it seemed advisable to use instead of the relative roughness $\frac{k}{r}$, its reciprocal $\frac{r}{k}$. Figure 9 shows to a logarithmic scale the relation of the resistance factor to the Reynolds number for the reciprocal values $\frac{r}{k}$ of the six relative roughnesses tested and for a smooth pipe (see tables 2 to 7). The bottom curve is for the smooth pipe. If the curve for $\lambda = f(Re)$ is studied for a given relative roughness, then it must be considered in three portions or ranges.

Within the first range, that of low Reynolds numbers, the roughness had no effect on the resistance, and for all values of $\frac{r}{k}$ the curve $\lambda = f(Re)$ coincides with the curve for the smooth pipe. This range includes all laminar flow and some turbulent flow. The portion of turbulent flow included increases as the relative roughness decreases. As long as laminar flow exists, the resistance factor may be expressed as:

$$\lambda = \frac{64}{Re} \tag{2}$$

This is represented in figure 9 by a straight line of slope 1:1. Within the first portion of turbulent flow in smooth pipes for a Reynolds number up to about $Re = 10^5$ the Blasius Resistance Law (reference 18) hol[illegible]

$$\lambda = \frac{0.316}{Re^{1/4}} \tag{3}$$

This is represented in the figure by a straight line of slope 1:4. The critical Reynolds number for all degrees of relative roughness occurs at about the same position as for the smooth pipe, that is, between 2160 and 2500.

Within the second range, which will be termed the transition range, the influence of the roughness becomes noticeable in an increasing degree; the resistance factor λ increases with an increasing Reynolds number. This transition range is particularly characterized by the fact that the resistance factor depends upon the Reynolds number as well as upon the relative roughness.

Within the third range the resistance factor is independent of the Reynolds number and the curves $\lambda = f(Re)$ become parallel to the horizontal axis. This is the range within which the quadratic law of resistance obtains.

The three ranges of the curves $\lambda = f(Re)$ may be physically interpreted as follows. In the first range the thickness δ of the laminar boundary layer, which is known to decrease with an increasing Reynolds number, is still larger than the average projection $(\delta > k)$. Therefore energy losses due to roughness are no greater than those for the smooth pipe.

In the second range the thickness of the boundary layer is of the same magnitude as the average projection $(\delta \approx k)$. Individual projections extend through the boundary layer and cause vortices which produce an additional loss of energy. As the Reynolds number increases, an increasing number of projections pass through the laminar boundary layer because of the reduction in its thickness. The additional energy loss than becomes greater as the Reynolds number increases. This is expressed by the rise of the curves $\lambda = f(Re)$ within this range.

Finally, in the third range the thickness of the boundary layer has become so small that all projections extend through it. The energy loss due to the vortices has now attained a constant value and an increase in the Reynolds number no longer increases the resistance.

The relationships within the third range are very simple. Here the resistance factor is independent of the Reynolds number and depends only upon the relative roughness. This dependency may be expressed by the formula

$$\lambda = \frac{1}{\left(1.74 + 2 \log \frac{r}{k}\right)^2} \tag{4}$$

In order to check this formula experimentally the value $\frac{1}{\sqrt{\lambda}}$ was plotted in figure 10 against $\log \frac{r}{k}$ and it was found that through these points there could be passed a line

$$\frac{1}{\sqrt{\lambda}} = 1.74 + 2 \log \frac{r}{k} \tag{5}$$

The entire field of Reynolds numbers investigated was covered by plotting the term $\frac{1}{\sqrt{\lambda}} - 2 \log \frac{r}{k}$ against $\log \frac{v_* k}{\nu}$. This term is particularly suitable dimensionally since it has characteristic values for conditions along the surface. The more convenient value $\log Re \sqrt{\lambda} - \log \frac{r}{k}$ might be used instead of $\log \frac{v_* k}{\nu}$, as may be seen from the following consideration. From the formula for the resistance factor

$$\lambda = \frac{dp}{dx} \frac{4r}{\rho \bar{u}^2} \tag{1}$$

the relationship between the shearing stress τ_o and the friction factor λ may be obtained. In accordance with the requirements of equilibrium for a fluid cylinder of length dx and radius r,

$$2\pi r \tau_o = \frac{dp}{dx} \pi r^2$$

or from equation (1)

$$\frac{\tau_o}{\rho} = \lambda \frac{\bar{u}^2}{8} \tag{6a}$$

or

$$\sqrt{\lambda} = 2.83 \frac{v_*}{\bar{u}} \tag{6b}$$

in which $v_* = \sqrt{\frac{\tau_o}{\rho}}$ is the friction velocity. There results

$$Re \sqrt{\lambda} = 5.66 \frac{v_* r}{\nu}$$

and

$$\log(\mathrm{Re}\,\sqrt{\lambda}) - \log\frac{r}{k} = \log\left(5.66\,\frac{v_*k}{\nu}\right) \tag{7a}$$

or

$$\log\frac{v_*k}{\nu} = \text{const} + \log(\mathrm{Re}\,\sqrt{\lambda}) - \log\frac{r}{k} \tag{7b}$$

From equation (5) there is obtained:

$$\frac{1}{\sqrt{\lambda}} - 2\log\frac{r}{k} = 1.74 \tag{5a}$$

It is evident then that the magnitude of $\left(\frac{1}{\sqrt{\lambda}} - 2\log\frac{r}{k}\right)$ is constant within the region of the quadratic law of resistance but within the other regions is variable depending on the Reynolds number. The preceding explains why the value $\log(\mathrm{Re}\sqrt{\lambda}) - \log\frac{r}{k}$ was used as the abscissa instead of $\log(\mathrm{Re}\sqrt{\lambda})$ as was done for the smooth pipe. Equation (5a) may now be written in the form

$$\frac{1}{\sqrt{\lambda}} - 2\log\frac{r}{k} = f\left(\log\frac{v_*k}{\nu}\right) \tag{8}$$

There occurs here, as the determining factor, the dimensionless term

$$\eta = \frac{v_*k}{\nu}$$

which is to be expected from the viewpoint of dimensional analysis. The relationship

$$\frac{1}{\sqrt{\lambda}} - 2\log\frac{r}{k} = f\left(\log\frac{v_*k}{\nu}\right)$$

as determined experimentally is shown in figure 11 (see tables 2 to 7) for five degrees of relative roughness. The sixth degree of relative roughness was not included because in that the assumption of geometrical similarity probably did not exist. It is evident that a smooth curve may be passed through all the plotted points.

The range I in which the resistance is unaffected by the roughness and in which all pipes have a behavior similar to that of a smooth pipe is expressed in this diagram (fig. 11) by the equation

$$\frac{1}{\sqrt{\lambda}} - 2 \log \frac{r}{k} = 0.8 + 2 \log \frac{v_* k}{\nu} \tag{9}$$

in which the value of a function f is determined by equation 8. The fact that the test points lie below this range is due to the influence of viscosity which is still present for these small Reynolds numbers. This indicates that the law expressed in equation 3 is not exactly fulfilled. The transition range, range II, is represented in figure 11 by a curve which at first rises, then has a constant value, and finally drops. The curves to be used in later computations will be approximated by three straight lines not shown (references 19 and 20) in figure 11. The range covered by the quadratic law of resistance, range III, in this diagram lies above $\log \frac{v_* k}{\nu} = 1.83$ and corresponds to equation (5a).

These lines may be expressed by equations of the form

$$\frac{1}{\sqrt{\lambda}} - 2 \log \frac{r}{k} = a + b \log \frac{v_* k}{\nu} \tag{10}$$

in which the constants a and b vary with $\frac{v_* k}{\nu}$ in the following manner:

$$\frac{1}{\sqrt{\lambda}} - 2 \log \frac{r}{k} = 1.18 + 1.13 \log \frac{v_* k}{\nu} \qquad \text{for} \quad 0.55 \leqq \log \frac{v_* k}{\nu} \leqq 0.85$$

$$= 2.14 \qquad \text{for} \quad 0.85 \leqq \log \frac{v_* k}{\nu} \leqq 1.15$$

$$= 2.81 - 0.588 \log \frac{v_* k}{\nu} \qquad \text{for} \quad 1.15 \leqq \log \frac{v_* k}{\nu} \leqq 1.83$$

It is clear that for each straight line

$$\lambda = \frac{1}{\left(a + b \log \frac{v_* k}{\nu} + 2 \log \frac{r}{k}\right)^2} \tag{11}$$

2. Velocity Distribution

Observations on velocity distributions were made for pipes with diameters of 2.5 centimeters, 5 centimeters, and 10 centimeters, with Reynolds numbers between 10^4 and 10^6 (see tables 8 to 13). Since the

velocity distributions were symmetrical, only one-half the curve had to be considered in the evaluation of test data. A dimensionless equation of the form

$$\frac{u}{U} = f\left(\frac{y}{r}\right) \tag{12}$$

was selected to show the variation of the velocity distribution with the value $\frac{r}{k}$. In this equation U is the maximum velocity, and u is the velocity at any point y distant from the wall in a pipe of radius r. This relationship is shown in figure 12 for a smooth pipe and for such velocity distributions at various degrees of relative roughness as lie within the region of the quadratic law of resistance. This figure indicates that as the relative roughness increases, the velocity distribution assumes a more pointed form. Our earlier tests with the smooth pipe have shown, however, that as the Reynolds number increases the velocity distribution assumes a more blunt form.

A very simple law for the velocity distribution in rough pipes is obtained from the following plotting. The dimensionless velocity $\frac{u}{v_*}$ is shown in figure 13 plotted against $\frac{y}{r}$. The term v_* is the "friction velocity," $v_* = \sqrt{\frac{\tau_0}{\rho}}$ as previously introduced. This figure indicates that in the regions away from the wall the velocity distributions are similar. If, in accordance with Von Kármán, the plotting is for $\frac{U - u}{v_*} = f\left(\frac{y}{r}\right)$, the similar curves merge to form a single curve (fig. 17). The velocity distributions for the different degrees of relative roughness also merge to almost a single curve if the dimensionless term $\frac{u}{v_*}$ is plotted against $\log \frac{y}{k}$. It may be seen that all the observed points agree very well with the straight line, only however for those velocity distributions which come within the region of the quadratic law of resistance (fig. 14). This line has the equation

$$\frac{u}{v_*} = 8.48 + 5.75 \log \frac{y}{k} = A + B \log \frac{y}{k} \tag{13}$$

Following the method of Prandtl (reference 21) in obtaining a universal law of velocity distribution in smooth pipes there is used here a dimensionless distance from the wall $\eta = y \frac{v_*}{\nu}$ to obtain the universal equation for velocity distribution

$$\frac{u}{v_*} = \varphi = 5.5 + 5.75 \log \eta \qquad (14)$$

If the relationship $\varphi = f(\log \eta)$ is now plotted for rough pipes, figures 15(a) to 15(f) are obtained, which in every case yield a straight line for the dimensionless velocity. Each figure corresponds to a definite relative roughness and to the several Reynolds numbers recorded; figure 15(a) corresponds to the smallest roughness $\frac{r}{k} = 507$, figure 15(b) to the next to smallest, etc. There is furthermore shown on every figure the velocity distribution in the smooth pipe as given by equation 14. The observation points lying on this straight line were obtained not in a smooth pipe but in a rough pipe at such a small Reynolds number that the influence of the roughness is not noticeable. These straight lines for a given relative roughness shift with an increasing Reynolds number to a position parallel to that of the straight line for the smooth pipe. A careful study of the individual test points shows that those near the wall (small values of $\log \eta$) as well as those near the axis (large values of $\log \eta$) lie slightly above the line.

The term A as indicated by equation (13) has a constant value in the region of the quadratic law of resistance. In the transition regions I and II, however, A depends upon the Reynolds number $Re = \frac{\bar{u}2r}{\nu}$ and on the relative roughness $\frac{k}{r}$ in such a manner that A essentially depends only on the product $Re\sqrt{\lambda}\,\frac{k}{r}$ in accordance with equation (7a). From equation (6b)

$$\sqrt{\lambda} = 2.83\,\frac{v_*}{\bar{u}}$$

so that

$$Re\sqrt{\lambda}\,\frac{k}{r} = 5.66\,\frac{v_* k}{\nu}$$

There may then be obtained an expression of the form

$$A = \frac{u}{v_*} - 5.75 \log \frac{y}{k} = f\left(\log \frac{v_* k}{\nu}\right) \qquad (15)$$

In order to determine the magnitude of A for each velocity distribution curve, the term $\frac{u}{v_*} - 5.75 \log \frac{y}{k}$ was obtained from figures 15(a) to 15(e) for every test point of each velocity curve and was plotted as a function of $\frac{y}{r}$. From the plotted result the value of A was determined for the

velocity curve under consideration. Particular care must be used in this determination at medium distances from the wall, since, on the one hand, the value of y cannot be accurately obtained for points near the wall, and furthermore the viscosity has a noticeable influence here, and on the other hand, a regular deviation always occurs for points near the axis. The value of A as found in this manner for all velocity curves was then plotted as a function of $\log \frac{v_* k}{\nu}$ (see fig. 16). The form of curve A as a function of $\log \frac{v_* k}{\nu}$ is very similar to the curve for the resistance law obtained by plotting $\frac{1}{\sqrt{\lambda}} - 2 \log \frac{r}{k}$ against $\log \frac{v_* k}{\nu}$ from equation (8).

Analytical proof of this relationship may be obtained by the same method as that used for the smooth pipe (references 5 and 21). In accordance with equation (13)

$$\frac{u}{v_*} = A + B \log \frac{y}{k} \qquad (16a)$$

or, if this equation is written for the pipe axis, that is, $u = U$, $y = r$:

$$\frac{U}{v_*} = A + B \log \frac{r}{k} \qquad (16b)$$

From the equation $\frac{U - u}{v_*} = f \frac{y}{r}$ there may be obtained by integration the term

$$\frac{U - \bar{u}}{v_*} = \beta \qquad (17a)$$

If $\frac{U - u}{v_*}$ is plotted as a function of $\left(\frac{y}{r}\right)^2$, the result will be

$$\beta = 3.75$$

Then, from equation (17a)

$$U = \bar{u} + \beta v_* \qquad (17b)$$

and from equation (6b) the relationship between the coefficient of resistance and the average velocity u is found from

$$\bar{u} = \frac{2.83}{\sqrt{\lambda}} v_* \qquad (18)$$

Substituting equation (18) into equation (17b) and dividing by v_*

$$\frac{U}{v_*} = \frac{2.83}{\sqrt{\lambda}} + \beta$$

and then from equation (16b)

$$\frac{2.83}{\sqrt{\lambda}} = A + B \log \frac{r}{k} - \beta \tag{19a}$$

or with $B = 5.75$

$$\frac{2.83}{\sqrt{\lambda}} - \left(5.75 \log \frac{r}{k} - \beta\right) = A \tag{19b}$$

The desired relationship between the velocity distribution and the law of resistance is given in equations (15) and (19b). It may be expressed in the following form

$$\frac{u}{v_*} - 5.75 \log \frac{y}{k} = \frac{2.83}{\sqrt{\lambda}} - \left(5.75 \log \frac{r}{k} - \beta\right) = f\left(\log \frac{v_* k}{\nu}\right) \tag{20}$$

Figure 16 contains in addition to the values of A computed from the velocity distributions by equation (15), the computed values obtained from the law of resistance by equation (19b). The agreement between the values of A determined by these two methods is satisfactory.

By the same method as in figure 11, the curve A may be represented as a function of $\log \frac{v_* k}{\nu}$. Within the range of the law of resistance where the effect of viscosity is not yet present the law for smooth pipes applies, that is,

$$0 \leqq \log \frac{v_* k}{\nu} \leqq 0.55 \qquad A = 5.5 + 5.75 \log \frac{v_* k}{\nu} \tag{21a}$$

The transition region from the law of resistance of the smooth pipe to the quadratic law of resistance of the rough pipe may be divided into three zones:

$$\text{I. } 0.55 \leqq \log \frac{v_* k}{\nu} \leqq 0.85 \qquad A = 6.59 + 3.5 \log \frac{v_* k}{\nu} \tag{21b}$$

$$\text{II. } 0.85 \leqq \log \frac{v_* k}{\nu} \leqq 1.15 \qquad A = 9.58 \tag{21c}$$

$$\text{III. } 1.15 \leqq \log \frac{v_* k}{\nu} \leqq 1.83 \qquad A = 11.5 - 1.62 \log \frac{v_* k}{\nu} \tag{21d}$$

and within the zone of the quadratic law of resistance:

$$\log \frac{v_* k}{\nu} > 1.83 \qquad A = 8.48 \tag{21e}$$

These expressions describe with sufficient accuracy the laws of velocity distribution and of resistance for pipes with walls roughened in the manner here considered.

Finally, it will be shown briefly that the Von Kármán (reference 2) equation for the velocity distribution

$$\frac{U - u}{v_*} = -\frac{1}{\kappa}\left[\ln\left(1 - \sqrt{1 - \frac{y}{r}}\right) + \sqrt{1 - \frac{y}{r}}\right] \tag{22}$$

derived analytically on the basis of his hypothesis of similarity, agrees with the experimental data. The term κ is a universal constant obtained from the velocity distribution. In figure 17, the curve drawn through the experimental points agrees almost exactly with the curve for this equation. With very large Reynolds numbers where the influence of viscosity is very slight the velocity distributions according to Von Karman's treatment do not depend upon the type of wall surface nor upon the Reynolds number. Good agreement with $\kappa = 0.36$ is obtained between experimental and theoretical curves for such velocity distribution up to the vicinity of the wall. It may be concluded from this that at a definite interval y, from the wall, the type of flow and the momentum change are independent of the type of wall surface.

In order to include those observation points for velocity distributions which are near the wall the term $\frac{U - u}{v_*}$ was evaluated from the universal velocity distribution equation (14) in the following manner: If equation (14) is written for the maximum velocity by letting $u = U$ and $y = r$, then

$$\frac{U}{v_*} = 5.5 + 5.75 \log \frac{v_* r}{\nu}$$

If equation (14) is subtracted from this equation, there is obtained

$$\frac{U - u}{v_*} = 5.75 \log \frac{r}{y} \tag{23}$$

In contrast to the theoretical curve of Von Kármán which agrees with the observations taken near the wall only if a different value of κ is used, the above equation obtained from the observations describes the entire range between the surface and the axis of the pipe. It is of

interest to consider for comparison the equation which Darcy (reference 6) obtained in 1855, on the basis of careful measurements. His equation for velocity distribution, in the notation of this article, is

$$\frac{U - u}{v_*} = 5.08\left(1 - \frac{y}{r}\right)^{3/2} \qquad (24)$$

In figure 17, equation (23) is represented by a full line and equation (24) by a dotted line. The Darcy curve shows good agreement except for points near the wall where $\frac{y}{r} < 0.35$. This imperfection of the Darcy formula is due to the fact that his observations nearest the wall were for $\frac{y}{r} = 0.33$. Up to this limit the agreement of equation (24) with the observed data is very good.

3. Exponential Law

Even though the velocity distribution is adequately described by equation (13) or equation (23), it is sometimes convenient to have an exponential expression which may be used as an approximation. Prandtl from a dimensional approach concluded from the Blasius law of resistance that the velocity u near the wall during turbulent flow varies with the 1/7 power of the distance from the wall, (references 22, 23, and 24), that is

$$u = ay^{1/7} \qquad (25)$$

in which a is a constant for each velocity curve. It is to be emphasized that the exponent 1/7 holds only for smooth pipes in the range of the Blasius law (up to $Re = 10^5$), but that for larger Reynolds numbers it decreases, as shown by our earlier observations, (references 5 and 25) to 1/10. The situation is entirely different in the case of rough pipes; here within the range of our experiments the exponent for an increasing relative roughness increases from 1/7 to 1/4.

Equation (25) may be written in another form if the velocity and the distance from the wall are made dimensionless by using the friction velocity v_*:

$$\frac{u}{v_*} = \varphi = C\left(\frac{yv_*}{\nu}\right)^n = C\eta^n$$

in which, according to equation (25), $n = 1/7$. Then

$$\log \varphi = \log C + n \log \eta \qquad (26)$$

If $\log \varphi$ is plotted as a function of $\log \eta$ there results a straight line with slope n. This relationship is shown in figure 18 for various degrees of relative roughness and also for a velocity distribution in a smooth pipe. All of the velocity distributions for rough pipes shown in this figure lie within the range of the quadratic law of resistance. It is evident from the figure that within the range of relative roughness investigated here the exponent n increases from 0.133 to 0.238. From the recorded curve for the smooth pipe $n = 0.116$. In order to determine the variation in the exponent n with the Reynolds number for a fixed relative roughness, the value of $\log \varphi$ as a function of $\log \eta$ has been determined for various Reynolds numbers and for a relative roughness $\frac{r}{k} = 126$. The change of slope of the line was found to be very slight with variations of Reynolds number. The smallest recorded values of Reynolds number lie within the region defined as range I of the resistance law where the coefficient of resistance λ is the same as for a smooth pipe; the next larger Reynolds numbers lie in range II (transition region), and the largest in range III (quadratic law of resistance). Figure 18 shows that points on the pipe axis deviate from the locations obtained by the exponential law.

4. Prandtl's Mixing Length

The well-known expression of Prandtl (references 1, 26, 27, and 28) for the turbulent shearing stress is:

$$\frac{\tau}{\rho} = l^2 \left|\frac{du}{dy}\right| \frac{du}{dy} \tag{27a}$$

The determination of the mixing length from the velocity profiles may be easily carried out by means of equation (27a). By rearrangement:

$$l = \sqrt{\frac{\frac{\tau}{\rho}}{\left(\frac{du}{dy}\right)^2}} \tag{27b}$$

The shearing stress τ at any point is in linear relationship to the shearing stress τ_o at the wall;

$$\tau = \tau_o\left(1 - \frac{y}{r}\right) \tag{28}$$

In the computation of the variation of mixing length with the distance from the wall by equation (27b), the value of $\frac{du}{dy}$ was found graphically

from the velocity distributions. This is somewhat difficult in the vicinity of the pipe axis since there the values of both $\frac{\tau}{\rho}$ and $\frac{du}{dy}$ are very small. The procedure necessary to obtain the value of l as accurately as possible has been described in detail in a previous article (reference 5).

The dimensionless mixing length distribution arrived at in this manner for large Reynolds numbers lying within the range of the quadratic law of resistance has been plotted in figure 19. The curve shown is that obtained from observations on smooth pipes, expressed according to Prandtl in the form:

$$\frac{l}{r} = 0.14 - 0.08\left(1 - \frac{y}{r}\right)^2 - 0.06\left(1 - \frac{y}{r}\right)^4 \qquad (29)$$

There exists, therefore, the same mixing length distribution in rough as in smooth pipes. This fact leads to the conclusion that the mechanics of turbulence, except for a thin layer at the wall, are independent of the type of wall surface.

In order to present in a compact form the variation of the mixing length distribution with the Reynolds number and with the relative roughness, there is plotted in figure 20 the term $\log\left(10 \frac{l}{y}\right)$ against the term $\log \eta = \log \frac{v_* y}{\nu}$. Each of the curves drawn from the top to the bottom of the figure corresponds to a given Reynolds number which is indicated as a parameter. Since $\frac{l}{y}$ has its largest values near the walls, the points for that region are in the upper part of the figure and points near the pipe axis are in the lower part. The curves drawn from left to right connect points of equal $\frac{y}{r}$-value. These curves are parallel to the horizontal axis for Reynolds numbers and degrees of relative roughness at which the viscosity has no influence. This horizontal direction does not obtain for low Reynolds numbers and for low degrees of relative roughness; there is, therefore, a noticeable effect of viscosity in such ranges. The fact is again borne out by figures 19 and 20 that for high Reynolds numbers where viscosity has no influence the mixing length distribution and therefore the mechanics of turbulence are independent of the Reynolds number and of the relative roughness.

5. Relationship between Average and Maximum Velocities

From equation (16b):

$$U = v_*\left(A + B \log \frac{r}{k}\right) \qquad (16c)$$

then from equation (17b):

$$\bar{u} = v_*\left(A + B \log \frac{r}{k} - \beta\right) \tag{30}$$

in which B is a constant (B = 5.75) for all Reynolds numbers and for all degrees of relative roughness, while A is constant only within the range of the quadratic law of resistance and varies with $\frac{v_* k}{\nu}$ outside of that range, and β has the value 3.75. If equation (30) is divided by equation (16c);

$$\frac{\bar{u}}{U} = \frac{A + B \log \frac{r}{k} - \beta}{A + B \log \frac{r}{k}} \tag{31}$$

Previous study has shown that in accordance with equations (21a) to (21e) the term A is a function of $\frac{v_* k}{\nu}$. Then for a fixed value of the relative roughness $\frac{r}{k}$ there is obtained from equation (31) the relationship;

$$\frac{\bar{u}}{U} = f\left(\log \frac{v_* k}{\nu}\right) \tag{32}$$

This expression is shown in figure 21 with each curve representing a different relative roughness. The curves have been computed from equation (31) and the points (tables 2 to 7) are experimental observations.

SUMMARY

This study deals with the turbulent flow of fluids in rough pipes with various degrees of relative roughness $\frac{k}{r}$ (in which k is the average projection of the roughening and r is the radius of the pipe). The requirements of similitude have been met by using test pipes which were geometrically similar in form (including the roughening). The roughness was obtained by sand grains cemented to the walls. These had an approximately similar form and a corresponding diameter k. If $\frac{k}{r}$ is the same for two pipes, the pipes are geometrically similar with geometrically similar wall surfaces.

There remained to be determined whether in these two pipes for a given Reynolds number the resistance factor λ would be the same and whether the function $\lambda = f(Re)$ would yield a smooth curve.

There was further to be determined whether the velocity distributions for pipes with equal relative roughness $\frac{k}{r}$ are similar and how they vary with the Reynolds number. The measurements show that there is actually a function $\lambda = f(Re)$. The velocity distributions for a given relative roughness show a very slight dependence on the Reynolds number, but on the other hand, the form of the velocity distribution is more pronouncedly dependent on the relative roughness. As the relative roughness increases, the velocity distribution assumes a more pointed form. A study of the question whether the exponential law of Prandtl also applied to rough pipes showed that velocity distributions may be expressed by an exponential law of the form $u = \alpha y^n$ in which the value of n increases from 0.133 to 0.238, as the relative roughness increases.

Experimental data were obtained for six different degress of relative roughness with Reynolds numbers ranging from $Re = 10^4$ to 10^6. If flow conditions are considered divided into three ranges, the observations indicated the following characteristics for the law of resistance in each range.

In range I for small Reynolds numbers the resistance factor is the same for rough as for smooth pipes. The projections of the roughening lie entirely within the laminar layer for this range.

In range II (transition range) an increase in the resistance factor was observed for an increasing Reynolds number. The thickness of the laminar layer is here of the same order of magnitude as that of the projections.

In range III the resistance factor is independent of the Reynolds number (quadratic law of resistance). Here all the projections of the roughening extend through the laminar layer and the resistance factor λ is expressed by the simple formula

$$\lambda = \frac{1}{\left(1.74 + 2 \log \frac{r}{k}\right)^2} \tag{4}$$

If a single expression is desired to describe the resistance factor for all ranges, then for all of the test data $\left(\frac{1}{\sqrt{\lambda}} - 2 \log \frac{r}{k}\right)$ may be plotted against $\left(\log \frac{v_* k}{\nu}\right)$ in which $\left(v_* = \sqrt{\frac{\tau_0}{\rho}}\right)$. The resulting general

expression is:

$$\lambda = \frac{1}{\left(a + b \log \frac{v_* k}{\nu} + 2 \log \frac{r}{k}\right)^2} \tag{11}$$

in which the values of a and b are different for the different ranges.

The velocity distribution is given by the general expression;

$$\frac{u}{v_*} = A + B \log \frac{y}{k} \tag{16a}$$

in which $B = 5.75$ and $A = 8.48$ within the region of the quadratic law of resistance, and in the other regions depends also upon $\frac{v_* k}{\nu}$. The relationship between the velocity distribution law and the law of resistance is found to be:

$$\frac{u}{v_*} - 5.75 \log \frac{y}{k} = \frac{2.83}{\sqrt{\lambda}} - \left(5.75 \log \frac{r}{k} - \beta\right) = f\left(\log \frac{v_* k}{\nu}\right) \tag{20}$$

in which $\beta = 3.75$ as determined from the Von Kármán velocity distribution law

$$\frac{U - u}{v_*} = f\left(\frac{y}{r}\right)$$

Integration of the preceding equation yields;

$$\frac{U - \bar{u}}{v_*} = \beta \tag{17a}$$

and from this, by means of the velocity distribution law, the ratio of the average velocity $\bar{u}$ to the maximum velocity U may be plotted against $\frac{v_* k}{\nu}$.

Finally, the Prandtl mixing length formula

$$\frac{\tau}{\rho} = l^2 \left|\frac{du}{dy}\right| \frac{du}{dy} \tag{27a}$$

was used to obtain the variation of the mixing length l with the distance y from the wall. The following empirical equation resulted:

$$\frac{l}{r} = 0.14 - 0.08\left(1 - \frac{y}{r}\right)^2 - 0.06\left(1 - \frac{y}{r}\right)^4 \qquad (29)$$

This empirical equation is applicable only to large Reynolds numbers and to the entire range of the quadratic law of resistance, where viscosity has no influence.

Translated by
A. A. Brielmaier
Washington University
St. Louis, Missouri
April, 1937

REFERENCES

1. Prandtl, L.: Turbulenz und ihre Entstehung. Tokyo-Vortrag 1929, J. Aéronaut. Res. Inst., Tokyo Imperial University, Nr. 65, 1930.

2. Von Kármán, Th.: Mechanische Ähnlichkeit und Turbulenz. Göttinger Nachr., Math.-Phys. Klasse 1930 und Verh. d. 3. intern. Kongr. f. techn. Mech., Stockholm 1930 (Stokholm 1931), Bd. 1. (Available as NACA TM 611.)

3. Nikuradse, J.: Widerstandsgesetz und Geschwindigkeitsverteilung von turbulenten Wasserströmungen in glatten und rauhen Rohren. Verh. d. 3. intern. Kongr. f. techn. Mech., Stockholm 1930 (Stockholm 1931) Bd. 1, p. 239.

4. Prandtl, L.: Zur turbulenten Strömung in Rohren und längs Platten. Ergeb. d. Aerodyn. Versuchsanst. zu Göttingen, 4. Lief., 1932, p. 18.

5. Nikuradse, J.: Gesetzmässigkeiten der turbulenten Strömung in glatten Rohren. VDI-Forsch.-Heft 356, 1932.

6. Darcy, H.: Recherches expérimentales relatives au mouvement de l'eau dans les tuyaux. Mémoires à l'Academie d. Sciences de l'Institute impérial de France, Bd. 15, 1858, p. 141.

7. Bazin, H.: Expériences nouvelles sur la distributation des vitesses dans les tuyaux, Mémoires à l'Academie d. Sciences de l'Institute de France, Bd. 32, Nr. 6, 1902.

8. v. Mises, R.: Elemente der technischen Hydrodynamik. Leipzig, B. G. Teubner 1914.

9. Stanton, T. E.: The Mechanical Viscosity of Fluids. Proc. Roy. Soc. London (A), Bd. 85, 1911, p. 366.

10. Schiller, L.: Über den Strömungswiderstand von Rohren verschiedenen Querschnitts und Rauhigkeitsgrades. Z. angew. Math. Mech., Bd. 3, 1923, p. 2.

11. Hopf, L.: Die Messung der hydraulischen Rauhigkeit. Z. angew. Math. Mech. Bd. 3, 1923, p. 329.

12. Fromm, K.: Strömungswiderstand in rauhen Rohren. Z. angew. Math. Mech. Bd. 3, 1923, p. 339.

13. Fritsch, W.: Der Einfluss der Wandrauhigkeit auf die turbulente Geschwindigkeitsverteilung in Rinnen. Z. angew. Math. Mech. Bd. 8, 1928, p. 199.

14. Treer, M. F.: Der Widerstandsbeiwert bei turbulenten Strömungen durch rauhe Kanäle. Phys. Z. Bd. 9, 1929, p. 539.

15. Treer, M. F.: Die Geschwindigkeitsverteilungen bei gradlinigen turbulenten Strömungen. Phys. Z. Bd. 9, 1929, p. 542.

16. Kumbruch, H.: Messung strömender Luft mittels Staugeräten. Forsch.-Arb. Ing.-Wes. Heft 240, Berlin 1921. (Available as NACA TM 302.)

17. Hoffmann, A.: Der Verlust in 90°-Rohrkrümmern mit gleichbleibendem Kreisquerschnitt. Mitt. hydraul. Inst. T. H. München, published by D. Thoma, Heft 3, 1929, p. 55.

18. Blasius, H.: Das Ähnlichkeitsgesetz bei Reibungsvorgängen in Flüssigkeiten. Forsch.-Arb. Ing.-Wes. Heft 131, Berlin 1913.

19. Prandtl, L.: Reibungswiderstand. Hydrodynamische Probleme des Schiffsantriebs. Published by G. Kempf and E. Förster 1932, p. 87.

20. Prandtl, L.: Neuere Ergebnisse der Turbulenzforschung. Z. VDI Bd. 77, Nr. 5, 1933, p. 105. (Available as NACA TM 720.)

21. Prandtl, L.: Zur turbulenten Strömung in Rohren und langs Platten. Ergebn Aerodyn. Versuchsanstalt. Göttingen, 4. Lief, 1932, p. 18.

22. Prandtl, L.: Ergebnisse der Aerodyn. Versuchsanstalt Göttingen, 3. Lief, 1927, p. 1.

23. Von Kármán, Th.: Über laminare und turbulente Reibung. Z. angew. Math. Mech. Bd. 1, Nr. 4, 1921, p. 233. (Available as NACA TM 1092.)

24. Nikuradse, J.: Untersuchungen über die Geschwindigkeitsverteilung in turbulenten Strömungen. Forsch.-Arb. Ing.-Wes. Heft 281, Berlin 1926.

25. Nikuradse, J.: Über turbulente Wasserströmungen in geraden Rohren bei sehr grossen Reynoldsschen Zahlen. Vortrag a. d. Geb. d. Aerodyn. u verw. Geb., Aachen 1929, published by A. Gilles, L. Hopf, and Th. V. Kármán, Berlin, J. Springer 1930, p. 63.

26. Prandtl, J.: Bericht über Untersuchungen zur ausgebildeten Turbulenz. Z. angew Math. Mech. Bd. 5, Nr. 2, 1925, p. 136. (Available as NACA TM 1231.)

27. Prandtl, L.: Bericht über neuere Turbulenzforschung. Hydraulische Probleme, Berlin, VDI-Verlag 1926, p. 1.

28. Prandtl, L.: Über ausgebildete Turbulenz. Verh. 2. intern. Kongr. f. techn. Mech., Zürich 1927.

TABLE 1

DIMENSIONS OF TEST PIPES

d = inside diameter

l_e = approach length

l_I = measuring length I

l_{II} = measuring length II

l_a = outlet length

x = total length

$\frac{x}{d}$ = relative total length

d (mm)	l_e (mm)	l_I (mm)	l_{II} (mm)	l_a (mm)	x (mm)	$\frac{x}{d}$
25	750	500	500	50	1800	72
50	1495	1000	1000	75	3570	71.4
100	4000	1500	1000	550	7050	70.5

TABLE 2

$\bar{u}$ cm/s	ν cm^2/s	$\frac{dp}{dx}$ dyn/cm^3	log Re	log(100 λ)	$\frac{1}{\sqrt{\lambda}}$ - 2 log r/k	log $\frac{v_* k}{\nu}$	$\frac{2.83}{\sqrt{\lambda}}$ - C	$\frac{\bar{u}}{U}$
r/k ≐ 507								
k = 0.01 cm d = 9.94 cm log r/k = 2.705								
15.45	0.0118	0.000351	4.114	0.456	0.51	0.000	4.95	0.815
20.2	.0118	.000574	4.230	.438	.64	.000	5.35	.819
25.0	.0118	.000840	4.322	.417	.79	.083	5.75	.824
27.3	.0118	.000975	4.362	.407	.86	.117	5.95	.825
27.3	.0118	.000966	4.362	.403	.88	.114	6.02	.824
34.4	.0118	.001525	4.462	.381	1.05	.212	6.48	.825
36.8	.0118	.00167	4.491	.380	1.06	.236	6.55	.830
40.4	.0118	.00195	4.532	.366	1.16	.267	6.80	.829
44.0	.0118	.00230	4.568	.365	1.17	.310	6.87	.832
46.4	.0118	.00251	4.591	.356	1.24	.322	7.05	.832
50.0	.0118	.00285	4.623	.347	1.31	.348	7.25	.834
55.9	.0118	.00347	4.672	.333	1.41	.391	7.50	.836
58.5	.0118	.00372	4.690	.324	1.48	.407	7.72	.835
61.8	.0118	.00410	4.716	.320	1.52	.428	7.85	.838
69.0	.0118	.00496	4.763	.307	1.62	.470	8.05	.839
76.0	.0118	.00597	4.806	.303	1.65	.508	8.08	.842
84.4	.0118	.00718	4.851	.292	1.74	.549	8.45	.841
94.0	.0118	.00878	4.898	.286	1.79	.593	8.58	.844
103.5	.0118	.01087	4.940	.278	1.86	.638	8.78	.843
106.0	.0112	.01085	4.973	.274	1.89	.661	8.85	.845
114.0	.0112	.01255	5.009	.274	1.90	.694	8.89	.848
119.8	.0112	.01378	5.025	.272	1.92	.713	8.95	.845
126	.0112	.01515	5.049	.270	1.93	.733	8.97	.847
147	.0116	.0202	5.100	.262	2.00	.781	9.17	.846
162	.0116	.0245	5.143	.260	2.02	.829	9.25	.847
184	.0116	.0314	5.199	.255	2.05	.878	9.29	.849
201	.0116	.0372	5.236	.253	2.07	.919	9.36	.847
217	.0116	.0435	5.270	.255	2.06	.944	9.35	.849
223	.0116	.0458	5.281	.253	2.07	.959	9.36	.849
234	.0116	.0501	5.303	.250	2.10	.971	9.45	.846
248	.0116	.0565	5.326	.252	2.08	1.004	9.42	.851
287	.0120	.0760	5.377	.255	2.06	1.053	9.35	.847
325	.0120	.0975	5.430	.253	2.07	1.107	9.36	.849
375	.0120	.1310	5.493	.258	2.03	1.172	9.25	.849
412	.0120	.1585	5.534	.260	2.01	1.214	9.19	.846
445	.0118	.1850	5.574	.262	2.00	1.255	9.15	.848
481	.0118	.2320	5.608	.290	1.95	1.303	9.05	.845
516	.0120	.2560	5.630	.272	1.96	1.317	8.95	.848
551	.0118	.2920	5.668	.272	1.92	1.352	8.95	.846
607	.0118	.3540	5.709	.272	1.91	1.394	8.93	.848
602	.0105	.3520	5.756	.278	1.87	1.446	8.83	.845
655	.0105	.4190	5.792	.279	1.85	1.483	8.75	.846
720	.0105	.5100	5.833	.283	1.82	1.525	8.67	.844
798	.0091	.6340	5.940	.286	1.80	1.633	8.63	.846
845	.0091	.7100	5.965	.288	1.78	1.659	8.55	.843
835	.0086	.5400	5.929	.289	1.77	1.623	8.51	.844
779	.0086	.6050	5.954	.288	1.78	1.648	8.55	.845
840	.0086	.7000	5.987	.286	1.79	1.680	8.57	.845

$\bar{u}$ = average velocity
ν = kinematic viscosity
$\frac{dp}{dx}$ = pressure gradient
Re = $\frac{\bar{u}d}{\nu}$ = Reynolds number
d = 2r = diameter of pipe
q = dynamic pressure for average velocity
$\lambda = \frac{dp}{dx}\frac{d}{q}$ = resistance factor

k = average projection of roughness
$v_* = \sqrt{\frac{\tau_o}{\rho}}$ = "friction" velocity
τ_o = shearing stress at wall
ρ = density
U = maximum velocity
C = 5.75 log $\frac{r}{k}$ - β

TABLE 3

$\bar{u}$ cm/s	ν cm²/s	$\frac{dp}{dx}$ dyn/cm³	log Re	log(100 λ)	$\frac{1}{\sqrt{\lambda}}$ - 2 log r/k	log $\frac{v_* k}{\nu}$	$\frac{2.83}{\sqrt{\lambda}}$ - C	$\frac{u}{U}$
r/k = 252								
k = 0.01 cm				d = 4.94 cm	log r/k = 2.401			
43.4	0.0132	0.0055	4.210	0.4506	1.15	0.290	6.77	0.816
51.0	.0132	.00728	4.279	.4349	1.26	.352	7.10	.820
78.2	.0132	.01524	4.465	.3808	1.65	.513	8.21	.830
86.0	.0132	.01775	4.507	.3636	1.77	.545	8.55	.831
94.8	.0132	.0213	4.549	.3579	1.82	.584	8.68	.830
104.0	.0130	.0255	4.597	.3562	1.84	.630	8.75	.832
116	.0130	.0308	4.644	.3434	1.94	.672	9.01	.836
158	.0130	.0549	4.778	.3257	2.08	.798	9.42	.838
174	.0130	.0668	4.820	.3282	2.06	.840	9.36	.840
214	.0128	.1000	4.916	.3222	2.11	.934	9.54	.842
252	.0128	.1375	4.987	.3197	2.12	1.003	9.55	.841
296	.0128	.1900	5.057	.3210	2.11	1.073	9.53	.839
322	.0126	.2265	5.100	.3228	2.10	1.118	9.50	.837
382	.0126	.3160	5.173	.3197	2.12	1.190	9.55	.840
407	.0124	.365	5.210	.3276	2.06	1.229	9.38	.841
468	.0120	.490	5.283	.3322	2.03	1.307	9.25	.836
555	.0118	.702	5.366	.3416	1.94	1.391	9.03	.833
735	.0116	1.257	5.494	.3504	1.89	1.526	8.85	.833
664	.0086	1.037	5.580	.3562	1.85	1.615	8.75	.832
734	.0086	1.280	5.623	.3602	1.80	1.660	8.65	.832
879	.0086	1.850	5.702	.3636	1.78	1.740	8.57	.832
121	.0117	.0329	4.708	.3371	1.99	.732	9.15	.836
486	.0119	.530	5.305	.3328	2.03	1.328	9.25	.836
854	.0120	1.724	5.544	.3562	1.85	1.580	8.75	.832
1104	.0089	2.925	5.787	.3661	1.76	1.842	8.53	.834
k = 0.02 cm				d = 9.94 cm	log r/k = 2.401			
72.3	0.0128	0.0058	4.748	0.3335	2.02	0.769	9.25	0.836
95.5	.0128	.00986	4.869	.3228	2.10	.884	9.46	.840
116.0	.0128	.0144	4.954	.3210	2.12	.966	9.53	.839
175.5	.0128	.0331	5.134	.3210	2.12	1.146	9.53	.840
232	.0128	.0589	5.255	.3294	2.05	1.272	9.30	.838
309	.0118	.1080	5.415	.3434	1.94	1.458	9.02	.830
454	.0118	.2375	5.580	.3551	1.84	1.692	8.75	.806
666	.0118	.522	5.748	.3608	1.80	1.782	8.64	.832
833	.0118	.828	5.845	.3666	1.76	1.881	8.50	.831
697	.0091	.583	5.881	.3688	1.75	1.919	8.49	.831
770	.0091	.719	5.924	.3727	1.71	1.964	8.37	.831
850	.0091	.872	5.967	.3705	1.72	2.004	8.42	.830
880	.0089	.816	5.991	.3716	1.72	2.000	8.40	.841

$\bar{u}$ = average velocity
ν = kinematic viscosity
$\frac{dp}{dx}$ = pressure gradient
$Re = \frac{\bar{u}d}{\nu}$ = Reynolds number
d = 2r = diameter of pipe
$\bar{q}$ = dynamic pressure for average velocity
$\lambda = \frac{dp}{dx}\frac{d}{\bar{q}}$ = resistance factor

k = average projection of roughness
$v_* = \sqrt{\frac{\tau_0}{\rho}}$ = "friction" velocity
τ_0 = shearing stress at wall
ρ = density
U = maximum velocity
$C = 5.75 \log \frac{r}{k} - \beta$

TABLE 4

$\bar{u}$ cm/s	ν cm²/s	$\frac{dp}{dx}$ dyn/cm³	log Re	log(100 λ)	$\frac{1}{\sqrt{\lambda}}$ -2 log r/k	log $\frac{v_* k}{\nu}$	$\frac{2.83}{\sqrt{\lambda}}$ - C	$\frac{\bar{u}}{U}$
				r/k = 126				
		k = 0.01 cm		d = 2.474 cm	log r/k = 2.10			
22.8	0.0132	0.00422	3.630	0.594	0.85	0.08279	5.93	0.795
25.2	.0132	.00506	3.675	.588	.88	.12418	6.01	.790
27.7	.0132	.00598	3.715	.576	.94	.16047	6.20	.794
30.7	.0132	.00715	3.760	.566	1.01	.19700	6.39	.799
34.4	.0132	.00870	3.810	.552	1.10	.24055	6.61	.798
36.3	.0132	.00996	3.833	.564	1.02	.26951	6.41	.800
41.8	.0132	.01210	3.895	.532	1.27	.31175	7.15	.805
44.8	.0132	.01355	3.925	.515	1.33	.33686	7.30	.806
47.5	.0132	.01480	3.950	.503	1.40	.35603	7.50	.809
49.2	.0132	.01570	3.965	.498	1.43	.36922	7.60	.810
55.2	.0132	.0195	4.015	.491	1.48	.41497	7.70	.810
68.8	.0132	.0289	4.111	.471	1.61	.50996	8.10	.816
83.7	.0132	.0408	4.196	.451	1.75	.57542	8.50	.818
98.2	.0132	.0532	4.265	.435	1.85	.63347	8.80	.818
114.0	.0132	.0713	4.330	.424	1.93	.69636	9.04	.824
129.5	.0132	.0900	4.386	.415	1.99	.74741	9.18	.825
136.5	.0132	.0990	4.425	.412	2.03	.75669	9.24	.823
157.5	.0132	.1287	4.470	.400	2.10	.82543	9.47	.824
167.0	.0132	.1432	4.496	.396	2.14	.84880	9.55	.829
173.0	.0132	.1550	4.511	.400	2.11	.86570	9.52	.828
189	.0133	.1823	4.550	.393	2.15	.90200	9.61	.825
223	.0132	.253	4.620	.392	2.16	.97267	9.64	.829
266	.0132	.360	4.697	.391	2.17	1.04844	9.65	.828
307	.0132	.488	4.760	.400	2.11	1.11428	9.53	.824
352	.0132	.646	4.820	.403	2.09	1.17609	9.46	.825
420	.0128	.930	4.910	.408	2.05	1.26811	9.30	.826
500	.0128	1.335	4.985	.414	2.01	1.34674	9.20	.823
590	.0128	1.896	5.057	.422	1.95	1.42259	9.04	.824
683	.0128	2.555	5.121	.424	1.93	1.48785	9.00	.825
755	.0128	3.164	5.164	.430	1.90	1.53656	8.92	.820
		k = 0.04 cm		d = 9.92 cm	log r/k = 2.10			
350	0.0089	0.175	5.591	0.450	1.75	1.96614	8.46	0.820
371	.0089	.201	5.616	.453	1.74	1.99739	8.42	.815
406	.0089	.238	5.655	.447	1.78	2.03383	8.55	.818
424	.0089	.261	5.675	.450	1.75	2.05346	8.47	.817
458	.0089	.301	5.708	.445	1.78	2.08600	8.56	.814
488	.0089	.347	5.736	.452	1.74	2.11661	8.45	.818
511	.0089	.374	5.756	.445	1.79	2.13194	8.58	.811
535	.0089	.410	5.775	.445	1.79	2.15259	8.58	.819
538	.0085	.420	5.798	.450	1.75	2.17754	8.48	.817
581	.0085	.490	5.831	.450	1.77	2.21005	8.51	.819
586	.0085	.494	5.835	.446	1.79	2.21245	8.58	.816
642	.0085	.598	5.874	.450	1.75	2.25310	8.48	.817
672	.0085	.650	5.894	.447	1.78	2.27184	8.55	.819
738	.0085	.791	5.935	.450	1.75	2.30963	8.48	.816
783	.0085	.877	5.961	.444	1.80	2.33746	8.60	.818
800	.0085	.927	5.970	.449	1.77	2.34928	8.51	.817
832	.0135	1.000	5.987	.447	1.78	2.16435	8.54	.818
121	.0117	.0200	4.950	.430	1.90	1.37694	8.92	.825
132	.0117	.0243	5.049	.432	1.88	1.41896	8.85	.821
124	.0117	.0206	5.021	.415	2.00	1.38346	9.18	.821
149	.0117	.0302	5.100	.422	1.95	1.46627	9.05	.823
159	.0117	.0347	5.130	.422	1.93	1.49665	9.00	.821
178	.0117	.0440	5.179	.430	1.90	1.54876	8.90	.823
185	.0117	.0475	5.196	.430	1.91	1.56467	8.92	.821
198	.0117	.0548	5.225	.435	1.87	1.59550	8.25	.822
198	.0117	.0544	5.225	.430	1.90	1.59329	8.90	.819
210	.0117	.0620	5.250	.436	1.85	1.62221	8.80	.822
222	.0117	.0696	5.274	.438	1.84	1.64738	8.76	.820
230	.0117	.0747	5.290	.438	1.84	1.69373	8.75	.822
181	.0088	.0460	5.310	.436	1.85	1.68124	8.77	.818
190	.0088	.0510	5.330	.439	1.83	1.70415	8.72	.820
199	.0088	.0560	5.350	.439	1.83	1.72428	8.70	.820
206	.0088	.0609	5.366	.444	1.80	1.74273	8.67	.818
219	.0088	.0687	5.393	.444	1.80	1.76938	8.62	.820
235	.0088	.0794	5.423	.446	1.79	1.80003	8.60	.820
244	.0088	.0857	5.432	.447	1.78	1.81690	8.60	.816
253	.0088	.0930	5.455	.450	1.76	1.83315	8.51	.818
265	.0088	.1025	5.476	.452	1.74	1.85491	8.48	.817
281	.0088	.1140	5.501	.447	1.78	1.87795	8.53	.819
301	.0081	.1300	5.525	.447	1.78	1.94300	8.53	.816
326	.0081	.1533	5.560	.450	1.76	1.98000	8.51	.818

$\bar{u}$ = average velocity

ν = kinematic viscosity

$\frac{dp}{dx}$ = pressure gradient

Re = $\frac{\bar{u}d}{\nu}$ = Reynolds number

d = 2r = diameter of pipe

$\bar{q}$ = dynamic pressure for average velocity

$\lambda = \frac{dp}{dx}\frac{d}{\bar{q}}$ = resistance factor

k = average projection of roughness

$v_* = \sqrt{\frac{\tau_0}{\rho}}$ = "friction" velocity

τ_0 = shearing stress at wall

ρ = density

U = maximum velocity

C = 5.75 log $\frac{r}{k}$ - β

TABLE 5

$\bar{u}$ cm/s	ν cm^2/s	$\frac{dp}{dx}$ dyn/cm^3	log Re	log(100 λ)	$\frac{1}{\sqrt{\lambda}}$ - 2 log r/k	log $\frac{v_* k}{\nu}$	$\frac{2.83}{\sqrt{\lambda}}$ - C	$\frac{\bar{u}}{U}$
r/k = 60								
k = 0.02 cm		d = 2.434 cm		log r/k = 1.78				
23.8	0.0128	0.00466	3.653	0.593	1.50	0.417	7.86	0.791
26.3	.0128	.00548	3.700	.577	1.59	.450	8.15	.795
28.9	.0128	.00650	3.740	.571	1.62	.529	8.19	.796
32.0	.0128	.00780	3.785	.560	1.69	.526	8.42	.798
37.5	.0128	.01030	3.851	.544	1.79	.588	8.68	.801
39.0	.0128	.01084	3.869	.531	1.83	.600	8.78	.804
42.7	.0128	.0124	3.909	.512	1.99	.627	9.27	.805
46.8	.0128	.0150	3.949	.512	1.99	.668	9.27	.802
52.0	.0128	.0182	3.996	.507	2.02	.711	9.35	.808
60.0	.0128	.0236	4.057	.494	2.10	.767	9.57	.808
64.6	.0128	.0270	4.090	.490	2.13	.797	9.66	.806
76.2	.0128	.0379	4.161	.494	2.10	.871	9.57	.813
90.4	.0128	.0526	4.236	.487	2.15	.942	9.73	.810
102.5	.0128	.0676	4.290	.487	2.15	.999	9.73	.811
129.0	.0128	.1055	4.391	.481	2.18	1.093	9.77	.814
135.6	.0128	.1190	4.412	.489	2.14	1.119	9.68	.810
171.0	.0128	.1890	4.512	.490	2.13	1.220	9.67	.810
182.5	.0128	.2142	4.540	.487	2.15	1.248	9.73	.811
188.0	.0128	.2334	4.553	.498	2.07	1.265	9.47	.808
187.0	.0120	.2280	4.580	.493	2.11	1.288	9.62	.810
200.0	.0120	.2690	4.609	.507	2.02	1.324	9.36	.807
214	.0118	.3062	4.654	.504	2.04	1.361	9.38	.810
224	.0118	.338	4.665	.507	2.02	1.380	9.35	.810
242	.0118	.397	4.699	.509	2.00	1.415	9.28	.808
262	.0116	.474	4.740	.517	1.95	1.462	9.17	.806
280	.0116	.544	4.769	.520	1.94	1.491	9.12	.807
302	.0114	.645	4.813	.528	1.89	1.537	8.97	.805
332	.0114	.777	4.849	.526	1.90	1.576	8.99	.805
399	.0114	1.165	4.930	.543	1.80	1.664	8.76	.804
421	.0114	1.270	4.954	.534	1.85	1.683	8.87	.801
508	.0114	1.890	5.034	.543	1.79	1.769	8.68	.803
671	.0114	3.30	5.155	.543	1.79	1.890	8.68	.802
566	.0114	2.36	5.083	.545	1.78	1.818	8.67	.799
717	.0114	3.83	5.185	.550	1.75	1.923	8.58	.800
795	.0114	4.57	5.231	.537	1.83	1.961	8.78	.802
k = 0.08 cm		d = 9.8 cm		log r/k = 1.78				
101	0.0132	0.0182	4.875	0.535	1.84	1.603	8.83	0.802
113	.0132	.0227	4.924	.534	1.85	1.651	8.87	.805
121	.0132	.0264	4.954	.542	1.80	1.685	8.76	.803
131	.0114	.0306	5.052	.535	1.84	1.780	8.86	.800
145	.0132	.0380	5.033	.540	1.81	1.763	8.77	.800
157	.0114	.0452	5.130	.545	1.78	1.865	8.67	.798
192	.0127	.0681	5.170	.550	1.75	1.906	8.58	.800
203	.0127	.0755	5.196	.547	1.77	1.928	9.56	.798
220	.0127	.0933	5.230	.568	1.64	1.965	8.27	.800
235	.0127	.1024	5.258	.551	1.74	1.996	8.57	.800
249	.0127	.1158	5.283	.555	1.71	2.021	8.47	.801
266	.0127	.1310	5.312	.551	1.74	2.049	8.57	.798
272	.0119	.1383	5.350	.555	1.71	2.089	8.47	.802
311	.0119	.1786	5.408	.550	1.75	2.143	8.58	.800
358	.0119	.2400	5.470	.555	1.72	2.207	8.48	.803
371	.0116	.2500	5.497	.543	1.69	2.228	8.68	.801
387	.0116	.278	5.515	.551	1.74	2.252	8.57	.799
418	.0116	.323	5.549	.550	1.75	2.286	8.58	.800
424	.0116	.338	5.554	.558	1.70	2.294	8.46	.799
445	.0116	.366	5.575	.551	1.74	2.311	8.57	.799
471	.0116	.411	5.600	.550	1.75	2.336	8.58	.801
495	.0116	.464	5.621	.560	1.69	2.362	8.42	.798
499	.0116	.454	5.625	.543	1.79	2.358	8.69	.804
514	.0115	.481	5.641	.543	1.79	2.375	8.69	.800
531	.0115	.521	5.655	.550	1.75	2.391	8.58	.798
535	.0115	.531	5.659	.551	1.74	2.396	8.57	.802
548	.0115	.567	5.668	.560	1.69	2.410	8.42	.798
576	.0115	.617	5.691	.553	1.73	2.428	8.55	.801
609	.0115	.689	5.714	.551	1.74	2.453	8.57	.800
656	.0115	.810	5.748	.558	1.70	2.488	8.46	.797
670	.0115	.830	5.757	.550	1.75	2.494	8.58	.802
721	.0115	.966	5.789	.551	1.74	2.526	8.57	.800
840	.0120	1.290	5.836	.547	1.77	2.570	8.65	.802
896	.0120	1.505	5.865	.555	1.72	2.603	8.49	.799
770	.0092	1.100	5.914	.553	1.73	2.648	8.54	.801
774	.0092	1.105	5.916	.550	1.75	2.658	8.58	.798
836	.0092	1.298	5.945	.551	1.74	2.686	8.57	.802
860	.0092	1.380	5.962	.555	1.71	2.699	8.47	.799

$\bar{u}$ = average velocity
ν = kinematic viscosity
$\frac{dp}{dx}$ = pressure gradient
$Re = \frac{\bar{u}d}{\nu}$ = Reynolds number
d = 2r = diameter of pipe
$\bar{q}$ = dynamic pressure for average velocity
$\lambda = \frac{dp}{dx}\frac{d}{\bar{q}}$ = resistance factor

k = average projection of roughness
$v_* = \sqrt{\frac{\tau_o}{\rho}}$ = "friction" velocity
τ_o = shearing stress at wall
ρ = density
U = maximum velocity
$C = 5.75 \log \frac{r}{k} - \beta$

TABLE 6

$\bar{u}$ cm/s	ν cm²/s	$\frac{dp}{dx}$ dyn/cm³	log Re	log(100 λ)	$\frac{1}{\sqrt{\lambda}}$ - 2 log r/k	log $\frac{v_* k}{\nu}$	$\frac{2.83}{\sqrt{\lambda}}$ - C	$\frac{\bar{u}}{U}$
				r/k = 30.6				
		k = 0.04 cm		d = 2.434 cm	log r/k = 1.486			
24.9	0.0129	0.00507	3.672	0.592	2.09	0.732	9.50	0.792
27.0	.0129	.00595	3.708	.590	2.10	.766	9.57	.794
29.6	.0129	.0072	3.748	.592	2.09	.807	9.54	.792
30.7	.0129	.0078	3.763	.597	2.06	.825	9.43	.793
32.3	.0129	.00839	3.785	.583	2.14	.839	9.67	.793
35.5	.0129	.0102	3.826	.585	2.13	.883	9.62	.794
39.2	.0129	.0126	3.869	.596	2.07	.929	9.66	.792
40.2	.0129	.0128	3.881	.578	2.17	.933	9.73	.797
45.0	.0129	.0161	3.929	.578	2.17	.934	9.74	.795
45.5	.0129	.0162	3.935	.583	2.14	.982	9.64	.795
48.0	.0123	.01835	3.978	.578	2.17	1.032	9.74	.796
51.6	.0123	.0214	4.009	.585	2.13	1.064	9.62	.797
56.6	.0123	.0258	4.049	.583	2.14	1.107	9.66	.795
60.8	.0123	.0303	4.079	.592	2.09	1.140	9.51	.792
67.4	.0123	.0370	4.124	.590	2.10	1.183	9.53	.795
68.4	.0123	.0390	4.130	.599	2.05	1.196	9.40	.791
78.5	.0123	.0514	4.190	.599	2.05	1.255	9.39	.791
94.2	.0123	.0756	4.270	.609	1.99	1.338	9.24	.790
98.7	.0123	.0840	4.290	.618	1.94	1.362	9.10	.788
103	.0123	.0912	4.309	.612	1.97	1.380	9.19	.790
202	.0128	.372	4.584	.639	1.82	1.667	8.72	.783
237	.0128	.519	4.653	.644	1.79	1.740	8.69	.782
300	.0116	.840	4.799	.647	1.78	1.888	8.62	.784
379	.0116	1.368	4.900	.656	1.73	1.993	8.49	.780
440	.0116	1.840	4.965	.656	1.73	2.057	8.49	.780
470	.0107	2.080	5.029	.652	1.75	2.120	8.55	.781
515	.0107	2.490	5.068	.650	1.70	2.158	8.41	.782
598	.0107	3.350	5.134	.650	1.76	2.223	8.58	.779
664	.0107	4.14	5.176	.650	1.76	2.270	8.57	.783
		k = 0.08 cm		d = 4.87 cm	log r/k = 1.486			
70.0	0.0128	0.0222	4.425	0.637	1.83	1.508	8.78	0.785
72.5	.0128	.0235	4.440	.630	1.78	1.519	8.89	.788
95.4	.0128	.0413	4.560	.637	1.83	1.642	8.79	.785
113.2	.0128	.0595	4.636	.647	1.78	1.721	8.63	.785
144	.0128	.0983	4.740	.654	1.74	1.826	8.51	.778
146	.0105	.1010	4.830	.654	1.74	1.922	8.51	.782
154	.0105	.1135	4.855	.661	1.70	1.947	8.41	.778
211	.0105	.212	4.990	.657	1.72	2.083	8.46	.778
272	.0105	.348	5.100	.652	1.74	2.190	8.54	.783
374	.0105	.663	5.240	.657	1.72	2.330	8.44	.782
406	.0105	.784	5.275	.657	1.72	2.367	8.45	.779
454	.0105	.958	5.323	.647	1.78	2.411	8.61	.784
640	.0105	1.945	5.473	.657	1.72	2.565	8.46	.780
975	.0105	4.470	5.655	.652	1.75	2.745	8.56	.783
		k = 0.16 cm		d = 9.64 cm	log r/k = 1.486			
99	0.0111	0.0235	4.934	0.656	1.73	2.032	8.48	0.783
135	.0111	.0436	5.068	.657	1.72	2.167	8.47	.779
171	.0111	.0706	5.170	.659	1.71	2.271	8.43	.781
193	.0111	.0903	5.223	.656	1.73	2.324	8.49	.779
207	.0111	.102	5.255	.652	1.75	2.350	8.55	.781
246	.0108	.146	5.342	.657	1.72	2.439	8.45	.780
248	.0108	.148	5.344	.657	1.72	2.441	8.47	.778
269	.0108	.175	5.394	.659	1.71	2.479	8.43	.779
300	.0108	.218	5.428	.659	1.71	2.526	8.43	.783
312	.0108	.236	5.444	.661	1.70	2.545	8.40	.779
368	.0108	.325	5.516	.657	1.72	2.614	8.48	.781
390	.0108	.367	5.541	.659	1.71	2.639	8.44	.778
406	.0108	.394	5.559	.657	1.72	2.655	8.48	.782
485	.0090	.568	5.776	.659	1.71	2.814	8.44	.779
603	.0090	.879	5.810	.659	1.71	2.909	8.45	.782
682	.0090	1.120	5.863	.657	1.72	2.961	8.47	.779
769	.0090	1.430	5.916	.659	1.71	3.014	8.43	.783
855	.0090	1.720	5.962	.650	1.76	3.054	8.57	.780
934	.0090	2.118	6.000	.659	1.71	3.100	8.45	.778

$\bar{u}$ = average velocity
ν = kinematic viscosity
$\frac{dp}{dx}$ = pressure gradient
Re = $\frac{\bar{u}d}{\nu}$ = Reynolds number
d = 2r = diameter of pipe
$\bar{q}$ = dynamic pressure for average velocity
$\lambda = \frac{dp}{dx}\frac{d}{\bar{q}}$ = resistance factor

k = average projection of roughness
$v_* = \sqrt{\frac{\tau_o}{\rho}}$ = "friction" velocity
τ_o = shearing stress at wall
ρ = density
U = maximum velocity
$C = 5.75 \log \frac{r}{k} - \beta$

TABLE 7

$\bar{u}$ cm/s	ν cm^2/s	$\frac{dp}{dx}$ dyn/cm^3	log Re	log(100 λ)	$\frac{1}{\sqrt{\lambda}}$ - 2 log r/k	log $\frac{v_* k}{\nu}$	$\frac{2.83}{\sqrt{\lambda}}$ - C	$\frac{\bar{u}}{U}$
				r/k = 15				
			k = 0.08 cm	d = 2.412 cm	log r/k = 1.176			
30.8	0.0126	0.00995	3.770	0.696	2.14	1.188	9.69	0.772
34.5	.0126	.01260	3.820	.699	2.13	1.239	9.66	.772
37.4	.0126	.01505	3.855	.707	2.09	1.276	9.57	.767
42.0	.0126	.01920	3.905	.712	2.06	1.317	9.46	.775
46.6	.0126	.02392	3.955	.717	2.04	1.377	9.40	.769
51.0	.0123	.02950	4.000	.730	1.97	1.435	9.23	.765
56.0	.0123	.03600	4.041	.734	1.94	1.477	9.14	.765
60.6	.0123	.04220	4.076	.736	1.94	1.511	9.13	.767
61.2	.0123	.0439	4.079	.744	1.90	1.520	9.03	.763
66.4	.0123	.0526	4.114	.751	1.87	1.559	8.93	.760
69.4	.0123	.0559	4.133	.740	1.92	1.572	9.06	.764
77.0	.0123	.0695	4.179	.744	1.92	1.619	9.06	.765
80.0	.0123	.0767	4.196	.754	1.85	1.641	8.89	.760
95.0	.0123	.1097	4.270	.760	1.82	1.718	8.80	.756
99.5	.0123	.1192	4.290	.756	1.84	1.737	8.85	.761
105.0	.0123	.1370	4.314	.769	1.78	1.767	8.69	.758
111.5	.0123	.1526	4.340	.763	1.80	1.791	8.74	.759
118.0	.0123	.1765	4.366	.778	1.74	1.822	8.54	.757
124.0	.0123	.1930	4.386	.772	1.77	1.841	8.64	.756
131.0	.0123	.2147	4.410	.772	1.77	1.865	8.64	.758
133.4	.0121	.2280	4.425	.782	1.72	1.884	8.49	.755
149.0	.0123	.282	4.466	.785	1.74	1.924	8.54	.755
169.0	.0123	.364	4.520	.780	1.73	1.979	8.53	.758
196.5	.0122	.493	4.590	.781	1.72	2.049	8.52	.755
214	.0121	.580	4.630	.777	1.75	2.087	8.56	.756
266	.0121	.900	4.725	.780	1.73	2.184	8.54	.756
325	.0120	1.350	4.811	.781	1.72	2.278	8.52	.753
364	.0120	1.680	4.865	.777	1.74	2.322	8.55	.755
375	.0118	1.776	4.885	.776	1.75	2.342	8.58	.756
447	.0117	2.540	4.965	.779	1.73	2.422	8.54	.756
484	.0117	2.982	5.000	.781	1.72	2.458	8.52	.754
532	.0117	3.611	5.042	.780	1.72	2.500	8.51	.754
560	.0108	4.019	5.098	.781	1.71	2.566	8.46	.752
640	.0108	5.100	5.155	.778	1.74	2.608	8.54	.756
675	.0108	5.809	5.179	.781	1.72	2.636	8.52	.756
788	.0098	7.900	5.285	.779	1.73	2.746	8.54	.755
			k = 0.16 cm	d = 4.82 cm	log r/k = 1.176			
75.5	0.0132	2.91	4.440	0.775	1.75	1.899	8.59	0.756
86.5	.0132	3.82	4.500	.777	1.75	1.957	8.55	.755
95.0	.0132	4.60	4.540	.778	1.73	1.998	8.54	.756
108.0	.0132	5.96	4.596	.780	1.72	2.055	8.53	.758
128.5	.0128	8.42	4.685	.781	1.72	2.144	8.52	.755
150.0	.0128	11.50	4.722	.777	1.75	2.210	8.55	.757
184.0	.0127	17.30	4.845	.775	1.75	2.300	8.59	.757
193.5	.0126	19.10	4.869	.778	1.73	2.327	8.54	.755
212	.0120	23.00	4.929	.780	1.72	2.391	8.51	.755
218	.0118	24.2	4.949	.779	1.73	2.409	8.54	.754
246	.0118	30.9	5.002	.777	1.74	2.460	8.53	.756
248	.0118	31.4	5.005	.775	1.75	2.464	8.59	.754
254	.0098	33.0	5.097	.778	1.73	2.555	8.54	.755
280	.0098	40.0	5.139	.783	1.72	2.599	8.49	.755
291	.0098	43.2	5.156	.784	1.71	2.618	8.47	.756
337	.0098	58.0	5.220	.777	1.74	2.677	8.53	.755
350	.0098	62.5	5.236	.780	1.73	2.695	8.54	.757
406	.0096	84.0	5.310	.778	1.73	2.767	8.54	.756
456	.0096	106.0	5.360	.775	1.75	2.816	8.59	.758
512	.0096	134.0	5.410	.780	1.72	2.870	8.51	.755
556	.0096	158.0	5.446	.780	1.73	2.906	8.54	.758
568	.0096	165.0	5.455	.777	1.75	2.914	8.55	.755
652	.0096	217.0	5.515	.781	1.72	2.976	8.51	.755
750	.0098	287.0	5.567	.778	1.74	3.030	8.54	.752
834	.0098	355.0	5.613	.780	1.73	3.073	8.51	.754
996	.0098	506.0	5.690	.784	1.71	3.152	8.47	.753
1018	.0072	530.0	5.834	.781	1.73	3.293	8.51	.755
1135	.0072	657.0	5.882	.777	1.75	3.338	8.55	.756
1360	.0072	944.0	5.959	.778	1.74	3.417	8.54	.754
1520	.0072	1180.0	6.008	.780	1.73	3.475	8.54	.750
976	.0076	497.0	5.793	.780	1.72	3.255	8.52	.755
1130	.0076	652.0	5.857	.777	1.74	3.314	8.53	.756
1342	.0076	918.0	5.930	.778	1.74	3.387	8.54	.758
1526	.0076	1190.0	5.987	.780	1.73	3.446	8.54	.754

$\bar{u}$ = average velocity
ν = kinematic viscosity
$\frac{dp}{dx}$ = pressure gradient
Re = $\frac{\bar{u}d}{\nu}$ = Reynolds number
d = 2r = diameter of pipe
$\bar{q}$ = dynamic pressure for average velocity
$\lambda = \frac{dp}{dx}\frac{d}{\bar{q}}$ = resistance factor

k = average projection of roughness
$v_* = \sqrt{\frac{\tau_o}{\rho}}$ = "friction" velocity
τ_o = shearing stress at wall
ρ = density
U = maximum velocity
C = 5.75 log $\frac{r}{k}$ - β

TABLE 8

r/k = 507

d cm	9.94	9.94	9.94	9.94	9.94	9.94	9.94
$\bar{u}$ cm/s	27	58.5	119.4	217	516	720	838
$10^2 \nu$ cm^2/s	1.18	1.18	1.14	1.16	1.20	1.05	0.86
10^{-3} Re	22.7	49.0	106	186	427	680	970
v_*	1.514	3.0	5.74	10.2	24.6	35.5	41.7
$\log \frac{v_* k}{\nu}$	0.106	0.398	0.702	0.936	1.303	1.53	1.686
$\frac{u}{v_*}$ - 5.75 log y/k	6.3	7.8	9.12	9.52	9.28	9.0	8.68

y/r	u cm/s	$\frac{du}{dy}$	u cm/s	$\frac{du}{dy}$	u cm/s	$\frac{du}{dy}$	u cm/s	$\frac{du}{dy}$	u cm/s	$\frac{du}{dy}$	u cm/s	$\frac{du}{dy}$	u cm/s	$\frac{du}{dy}$
0.00	14.8	-----	32.2	-----	58.0	------	98.0	------	265.0	-----	422	-----	523	------
.02	19.0	29.65	41.5	61.2	84.0	122.00	150	225	364	585	520	843	608	1022
.04	21.2	17.23	45.6	32.6	94.0	64.0	165	116	402	306	573	444	670	526
.07	23.0	9.46	49.7	19.0	101.3	38.0	180	69	432	178	625	256	725	309
.10	24.3	6.82	52.2	13.94	106.2	27.3	190	50	458	129	652	182	761	222
.15	25.8	4.88	55.1	10.00	112.0	19.7	201	36	487	90	689	128	803	153
.20	26.8	4.00	57.3	8.13	116.5	16.3	208	29.3	505	72	719	101	832	119
.30	28.3	2.80	60.5	5.79	123.0	11.4	220	20.8	531	51	758	72	874	84
.40	29.5	2.20	63.0	4.47	128.0	9.23	230	15.9	552	38.7	788	55	912	64
.50	30.4	1.80	64.8	3.56	132.0	7.26	237	13.1	568	31.8	812	45	940	52.5
.60	31.2	1.51	66.4	3.05	135.0	6.04	242.5	10.8	581	26.2	832	37	960	43.6
.70	31.8	1.21	67.7	2.47	137.6	4.80	247.5	8.75	591	21.4	848	30	978	35.5
.80	32.35	0.98	68.8	1.97	139.5	3.82	251.0	7.01	600	17.0	861	24	992	28.4
.90	32.75	.68	69.7	1.35	140.8	2.65	253.5	4.74	608	11.6	871	16.5	1003	19.2
.96	32.90	.60	70.1	.83	141.2	1.64	254.5	2.93	611.2	7.3	875	10.2	1008	12.0
.98	32.95	.42	70.2	.54	141.3	1.16	254.8	2.08	611.6	5.2	876	7.3	1010	8.7
1.00	33.0	-----	70.3	-----	141.4	------	255.0	------	612.0	-----	877	-----	1011	------

u = velocity at any point
y = distance from wall
Re = $\frac{\bar{u}d}{\nu}$ = Reynolds number
$\bar{u}$ = average velocity
d = diameter of pipe
ν = kinematic viscosity

$v_* = \sqrt{\frac{\tau_0}{\rho}}$ = "friction" velocity
τ_0 = shearing stress at wall
ρ = density
k = average projection of roughness
$\frac{r}{k}$ = relative roughness

TABLE 9

r/k = 252

d cm	4.924		4.924		4.924		4.924		4.924		4.924	
$\bar{u}$ cm/s	50.5		122		253.5		486		840		1127	
$10^2 \nu$ cm^2/s	1.17		1.17		1.205		1.19		1.20		0.89	
10^{-3} Re	21.4		51.0		103.5		202		344		624	
v_*	2.87		6.4		13.1		25.45		45.8		61.0	
$\log \frac{v_* k}{\nu}$	0.391		.728		1.032		1.321		1.573		1.826	
$\frac{u}{v_*}$ - 5.75 log y/k	7.7		9.10		8.56		9.3		8.9		8.6	
y/r	u cm/s	$\frac{du}{dy}$	u cm/s	$\frac{du}{dy}$	u cm/s	$\frac{du}{dy}$	u cm/s	$\frac{du}{dy}$	u cm/s	$\frac{du}{dy}$	u cm/s	$\frac{du}{dy}$
0.00	24	------	72.0	------	120	-----	210	------	328	------	532	----
.02	35	112	86.0	265	170	565	332	1145	593	2106	794	2954
.04	39	60	94.2	139	192	292	374	600	658	1111	875	1526
.07	42.7	35	101.5	83	209	172	410	346	717	648	951	895
.10	45.2	26	107.0	60	221	125	432	254	759	462	1000	643
.15	47.8	18	113.0	42	234	91	460	179	804	324	1062	443
.20	49.7	14.8	117.7	34	244	73	478	144	840	260	1107	346
.30	52.8	10.4	125.8	25	262	52	506	103	892	184	1178	247
.40	55.0	8.26	131.0	18.9	273	40	528	78	930	142	1225	188
.50	56.8	6.80	134.7	15.6	282	33	545	64	961	118	1266	154
.60	58.3	5.80	138.0	12.9	290	27	560	53	986	96	1303	127
.70	59.5	4.60	141.0	10.5	296	21.8	569	43	1006	78	1330	104
.80	60.5	3.70	143.3	8.2	301	17.2	578	34.5	1024	62	1350	83
.90	61.2	2.56	144.8	5.7	303.5	12.0	584	23.6	1036	42	1366	56
.96	61.5	1.56	145.6	3.54	304.8	7.4	588	14.6	1040	26	1369	35
.98	61.6	1.11	145.7	2.50	305.2	5.2	589.2	10.4	1042	18.7	1372	25
1.00	61.7	------	145.8	------	305.5	-----	590.0	------	1043	------	1373	----

u = velocity at any point
y = distance from wall
Re = $\frac{\bar{u}d}{\nu}$ = Reynolds number
$\bar{u}$ = average velocity
d = diameter of pipe
ν = kinematic viscosity

$v_* = \sqrt{\frac{\tau_o}{\rho}}$ = "friction" velocity
τ_o = shearing stress at wall
ρ = density
k = average projection of roughness
$\frac{r}{k}$ = relative roughness

TABLE 10

$r/k = 126$

d cm	2.474	2.474	2.474	2.474	9.92	9.92	9.92	9.92
u cm/s	81.8	117	238	530	205	374	575	820
$10^2 \nu$ cm^2/s	1.21	1.22	1.17	1.17	.88	.89	.89	.85
10^{-3} Re	16.7	23.7	50.5	112	231	417	640	960
v_*	4.90	6.7	13.25	30.8	12.0	22.28	34.3	49.0
$\log \frac{v_* k}{\nu}$	.606	.731	1.045	1.413	1.734	2.000	2.188	2.361
$\frac{u}{v_*} - 5.75 \log y/k$	8.75	9.14	9.55	9.35	8.75	8.50	8.35	8.38

y/r	u cm/s	$\frac{du}{dy}$	u cm/s	$\frac{du}{dy}$	u cm/s	$\frac{du}{dy}$	u cm/s	$\frac{du}{dy}$	u cm/s	$\frac{du}{dy}$	u cm/s	$\frac{du}{dy}$	u cm/s	$\frac{du}{dy}$	u cm/s	$\frac{du}{dy}$
0.00	47	-----	52.0	-----	109	------	262	----	84	-----	233	-----	352	-----	366	------
.02	59	370	79.0	527	159	1100	368	2660	135	271	250	516.0	383	794	557	1670
.04	66	196	89.4	277	181	570	416	1385	152	138	284	270	436	397	619	616
.07	71.1	119	98.4	164	199	340	454	818	167	82	312	158	476	237	676	380
.10	74.8	87	103.6	121	210	250	478	597	178	59	331	113	506	165	718	262
.15	79.8	61	110.0	87	223	179	513	425	189	41	350	80	536	120	760	181
.20	82.9	49	115.0	71	232	145	532	343	199	33.5	366	62	564	95	798	138
.30	88.0	35	121.8	49	245	103	565	243	211	23.7	390	44	597	66	852	96
.40	92.0	27	126.6	39	255	79	588	187	222	18.2	407	34	619	51	887	74
.50	95.2	23	130.3	32	263	66	606	154	229.5	14.9	419	28	640	42	916	60
.60	97.7	19.2	133.6	27	269.3	55	620	128	235.0	12.2	431	23	657	34	939	50
.70	99.6	15.7	136.4	21.7	275.0	44	633	104	240.5	10.0	439	19	667	27	959	41
.80	100.6	12.3	138.6	17.3	280.0	35	644	82	246.0	7.6	448	14.1	678	21	979	31
.90	101.4	8.6	140.7	12.1	284.0	24	652	55	248.4	5.5	453	10.3	690	15.2	988	22.5
.96	102.0	5.3	141.6	7.4	285.7	15	655	35	249.5	3.4	456	6.4	696	9.5	994	14.2
.98	102.1	3.7	141.8	5.2	286.2	10.6	656	25	249.8	2.4	457	4.6	698	6.7	996	10.0
1.00	102.2	-----	142.0	-----	286.8	------	657	----	250.0	-----	458	-----	701	-----	998	------

u = velocity at any point
y = distance from wall
Re = $\frac{\bar{u}d}{\nu}$ = Reynolds number
$\bar{u}$ = average velocity
d = diameter of pipe
ν = kinematic viscosity

$v_* = \frac{\tau_o}{\rho}$ = "friction" velocity
τ_o = shearing stress at wall
ρ = density
k = average projection of roughness
$\frac{r}{k}$ = relative roughness

TABLE 11

r/k = 60

d cm	2.434	2.434	2.434	2.434	9.8	9.8	9.8
u cm/s	76.2	147.6	330.0	544.0	309	514	774
$10^2 \nu$ cm^2/s	1.21	1.22	1.15	1.14	1.12	1.15	1.12
10^{-3} Re	15.3	29.5	70	116	271	438	677
v_*	4.75	9.22	21.6	36.2	20.72	34.4	51.9
$\log \frac{v_* k}{\nu}$	.903	1.180	1.574	1.809	2.164	2.378	2.568
$\frac{u}{v_*} - 5.75 \log y/k$	9.50	9.55	9.09	8.7	8.50	8.48	8.44

y/r	u cm/s	$\frac{du}{dy}$	u cm/s	$\frac{du}{dy}$	u cm/s	$\frac{du}{dy}$	u cm/s	$\frac{du}{dy}$	u cm/s	$\frac{du}{dy}$	u cm/s	$\frac{du}{dy}$	u cm/s	$\frac{du}{dy}$
0.00	34.0	-----	62	-----	125	------	297	----	152	-----	198	-----	259	-----
.02	50.6	369	95	760	211	3550	392	3140	193	471	316	812	475	1240
.04	57.0	189	110.5	390	250	950	436	1592	224	246	370	420	557	640
.07	62.5	115	121.0	238	277	575	464	950	245	143	411	246	624	373
.10	66.4	83	128.5	172	293	422	496	687	266	104	442	175	666	268
.15	71.0	59	137.0	123	314	300	520	487	286	74	474	125	714	189
.20	74.0	50	143.0	100	330	255	558	400	300	59	500	98	753	146
.30	79.0	34	153.0	70	352	171	585	283	323	42	536	70	793	105
.40	82.4	27	160.5	56	368	133	609	214	349	32	562	53	848	80
.50	85.0	22	166.0	45	381	109	626	179	353	26.4	582	44	879	65
.60	87.0	17.5	170.7	38	393	91	642	148	362	22.0	600	36.5	904	54
.70	89.0	14.9	174.5	30.7	402	73	653	120	370	18.0	616	31.0	925	43
.80	90.6	12.0	177.5	24.2	409	58	662	95	378	14.3	628	24.0	941	35
.90	92.2	8.3	180.0	16.8	416	40	665	65	384	9.6	636	16.0	955	24
.96	92.7	5.1	181.5	10.4	418	25	666	40	387	6.0	640	10.0	962	15
.98	92.8	3.6	182.0	7.2	419	17.4	666.5	28	387.5	4.3	641.4	7.1	964	10.7
1.00	93.0	-----	182.5	-----	419.5	------	667	----	388.0	-----	642.0	-----	966	------

u = velocity at any point
y = distance from wall
Re = $\frac{\bar{u}d}{\nu}$ = Reynolds number
$\bar{u}$ = average velocity
d = diameter of pipe
ν = kinematic viscosity

$v_* = \sqrt{\frac{\tau_o}{\rho}}$ = "friction" velocity
τ_o = shearing stress at wall
ρ = density
k = average projection of roughness
$\frac{r}{k}$ = relative roughness

TABLE 12

$r/k = 30.6$

d cm	2.434	2.434	2.434	2.434	4.87	4.87	9.64
$\bar{u}$ cm/s	61	116.5	217.5	459	420	796	734
$10^2 \nu$ cm^2/s	1.225	1.23	1.23	1.07	1.05	1.046	1.11
10^{-3} Re	12.1	23	43	104	195	372	638
v_*	4.25	8.3	16.2	34.8	31.9	60	55.7
$\log \frac{v_* k}{\nu}$	1.140	1.428	1.720	2.114	2.389	2.661	2.906
$\frac{u}{v_*} - 5.75 \log y/k$	9.6	9.16	8.7	8.50	8.48	8.42	8.50

y/r	u cm/s	$\frac{du}{dy}$	u cm/s	$\frac{du}{dy}$	u cm/s	$\frac{du}{dy}$	u cm/s	$\frac{du}{dy}$	u cm/s	$\frac{du}{dy}$	u cm/s	$\frac{du}{dy}$	u cm/s	$\frac{du}{dy}$
0.00	20.0	-----	38.0	-----	61	------	199	----	112	-----	245	----	225	-----
.02	36.5	332	67.5	680	128	1390	258	3300	215	1465	461	2880	430	1444
.04	43.2	171	80.9	345	156	706	328	1610	270	784	535	1518	500	728
.07	48.5	106	91.7	208	173	426	367	960	325	452	610	876	570	419
.10	51.5	74	98.5	152	185	310	395	696	351	326	660	628	616	302
.15	56.0	51.4	106.4	109	200	223	424	491	380	230	718	438	670	209
.20	59.0	43.0	113.0	89	211	181	447	401	404	185	766	344	715	162
.30	64.0	30.6	121.4	62	228	135	484	284	440	131	833	245	777	115
.40	67.4	23.8	128.0	49	242	99	509	219	467	100	880	188	819	88
.50	70.0	19.7	132.5	40.0	252	82	531	178	489	83	918	154	883	72
.60	72.0	16.8	136.5	33.6	261	67	551	148	507	68	950	126	881	59
.70	73.9	13.5	140.0	27.0	267	54	568	120	519	55	976	104	904	49
.80	75.6	10.8	142.6	21.5	272	43.4	580	95	530	44	994	83	925	39
.90	76.6	7.5	144.8	15.0	276	30.0	587	64	539	30	1008	57	940	26
.96	77.0	4.6	145.6	9.2	278	18.4	590	40	541	18.6	1016	35	948	16.5
.98	77.1	3.3	145.8	6.8	278.6	13.0	591	30	542	13.2	1017	25	951	11.7
1.00	77.2	-----	146.0	-----	279.0	------	592	----	543	------	1018	----	952	------

u = velocity at any point
y = distance from wall
Re = $\frac{\bar{u}d}{\nu}$ = Reynolds number
$\bar{u}$ = average velocity
d = diameter of pipe
ν = kinematic viscosity

$v_* = \sqrt{\frac{\tau_o}{\rho}}$ = "friction" velocity
τ_o = shearing stress at wall
ρ = density
k = average projection of roughness
$\frac{r}{k}$ = relative roughness

TABLE 13

$r/k = 15$

d cm	2.412		2.412		2.412		2.412		2.412		4.82	
$\bar{u}$ cm/s	57.2		112		215		524		956		873	
$10^2 \nu$ cm²/s	1.225		1.23		1.21		1.17		1.17		.98	
10^{-3} Re	11.3		22.2		43		108		197		430	
v_*	4.74		9.66		18.87		46.6		85		76.1	
$\log \frac{v_* k}{\nu}$	1.491		1.802		2.095		2.504		2.763		3.09	
$\frac{u}{v_*} - 5.75 \log y/k$	9.20		8.7		8.44		8.35		8.46		8.50	
y/r	u cm/s	$\frac{du}{dy}$	u cm/s	$\frac{du}{dy}$	u cm/s	$\frac{du}{dy}$	u cm/s	$\frac{du}{dy}$	u cm/s	$\frac{du}{dy}$	u cm/s	$\frac{du}{dy}$
0.00	17.0	-----	30.0	-----	62	------	170	----	302	----	205	----
.02	33.9	480	62.0	800	107	1630	270	4260	432	8000	450	3850
.04	39.6	180	73.0	420	136	856	320	2200	628	4235	549	1985
.07	44.6	120	86.0	248	158	500	367	1298	710	2440	643	1140
.10	48.5	81	94.0	181	174	370	406	940	767	1770	702	830
.15	52.9	61.6	102.5	129	191	265	456	667	846	1267	773	578
.20	56.2	47.8	109.5	104	206	217	492	544	910	976	830	457
.30	61.0	36.2	120.0	74	228	148	545	379	1006	699	917	321
.40	64.7	27.0	127.5	57	243	115	585	290	1080	530	978	246
.50	67.7	21.0	133.5	47	253	96	617	239	1143	429	1027	201
.60	70.0	16.2	138.5	40	263	79	647	199	1195	359	1070	166
.70	72.0	15.0	142.0	32	271	64	660	160	1237	290	1107	136
.80	73.8	12.4	145.5	25	277	52	689	128	1267	230	1137	108
.90	74.8	9.2	148.0	17.6	283	35.5	701	86	1290	160	1162	74
.96	75.6	7.4	149.5	10.8	285	22.0	704	54	1296	101	1172	47
.98	75.9	6.2	149.8	7.6	286	15.6	706	38	1298	72	1174	33
1.00	76.0	-----	150.0	-----	286.5	------	707	----	1300	----	1176	----

u = velocity at any point
y = distance from wall
$Re = \frac{\bar{u}d}{\nu}$ = Reynolds number
$\bar{u}$ = average velocity
d = diameter of pipe
ν = kinematic viscosity

$v_* = \sqrt{\frac{\tau_o}{\rho}}$ = "friction" velocity
τ_o = shearing stress at wall
ρ = density
k = average projection of roughness
$\frac{r}{k}$ = relative roughness

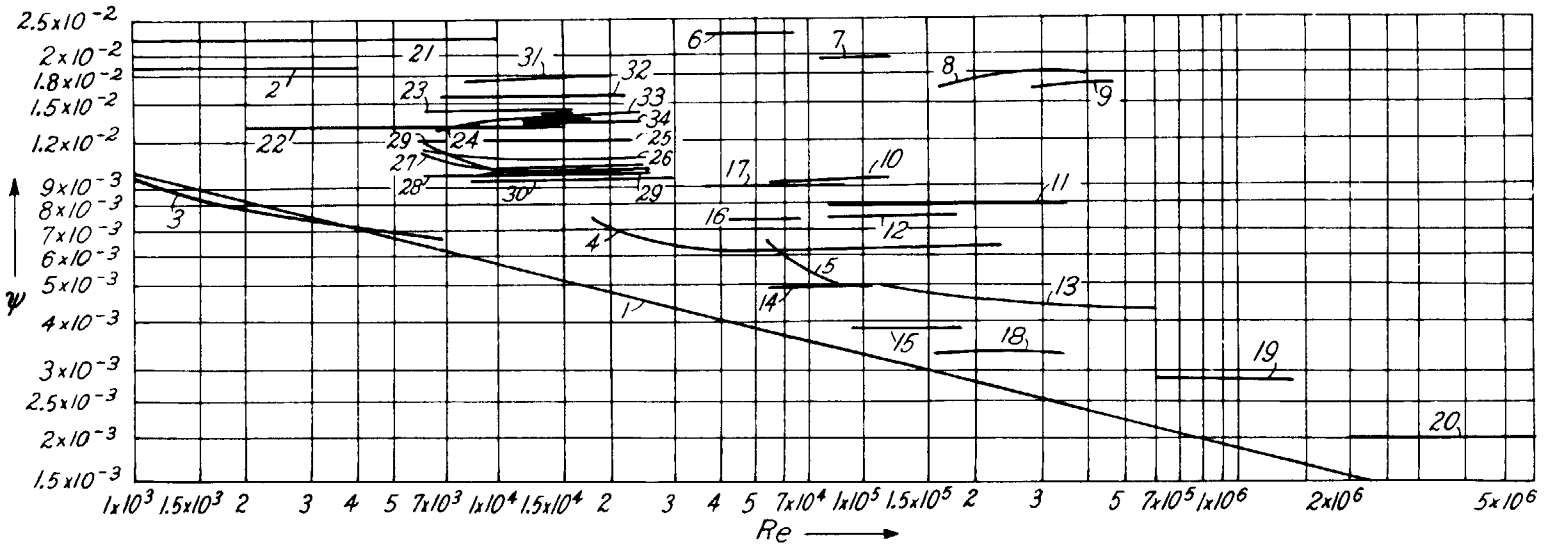

Figure 1.- Relation between the resistance factor $\psi = \frac{\lambda}{2}$ and the Reynolds number for surface roughness. (The numbers on the curves indicate the test results of various investigators.)

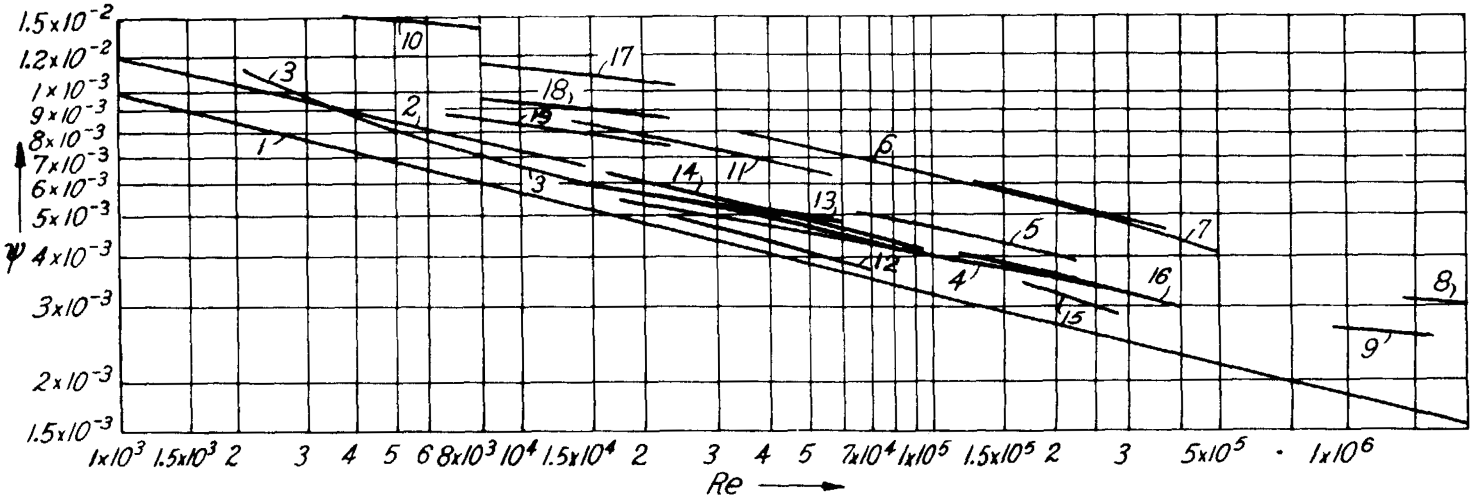

Figure 2.- Relation between the resistance factor $\psi = \frac{\lambda}{2}$ and the Reynolds number for surface corrugation. (The numbers on the curves indicate the test results of various investigators.)

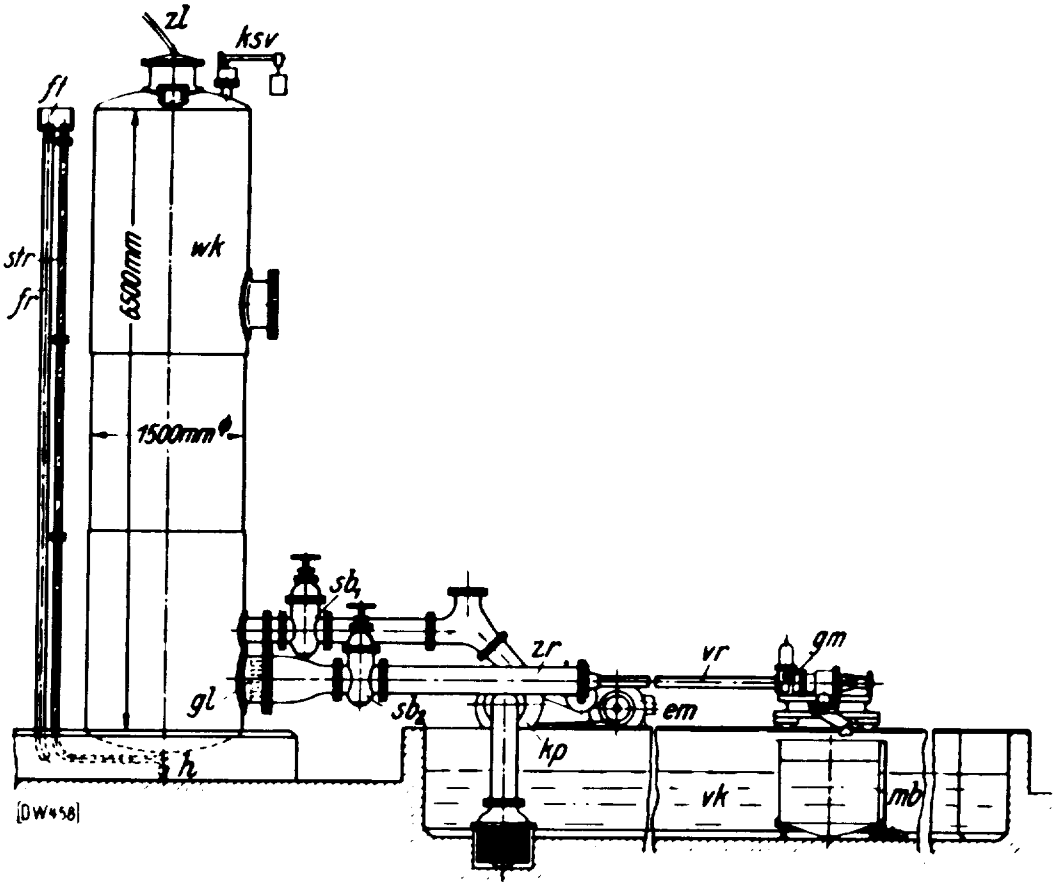

Figure 3.- Test apparatus.

em = electric motor	h = outlet valve
kp = centrifugal pump	zr = feed line
vk = supply canal	mb = measuring tank
wk = water tank	gm = velocity measuring device
vr = test pipe	ksv = safety valve on water tank
zl = supply line	sb_1 = gate valve between wk and kp
str = vertical pipe	sb_2 = gate valve between wk and zr
fr = overflow pipe	
ft = trap	gl = baffles for equalizing flow

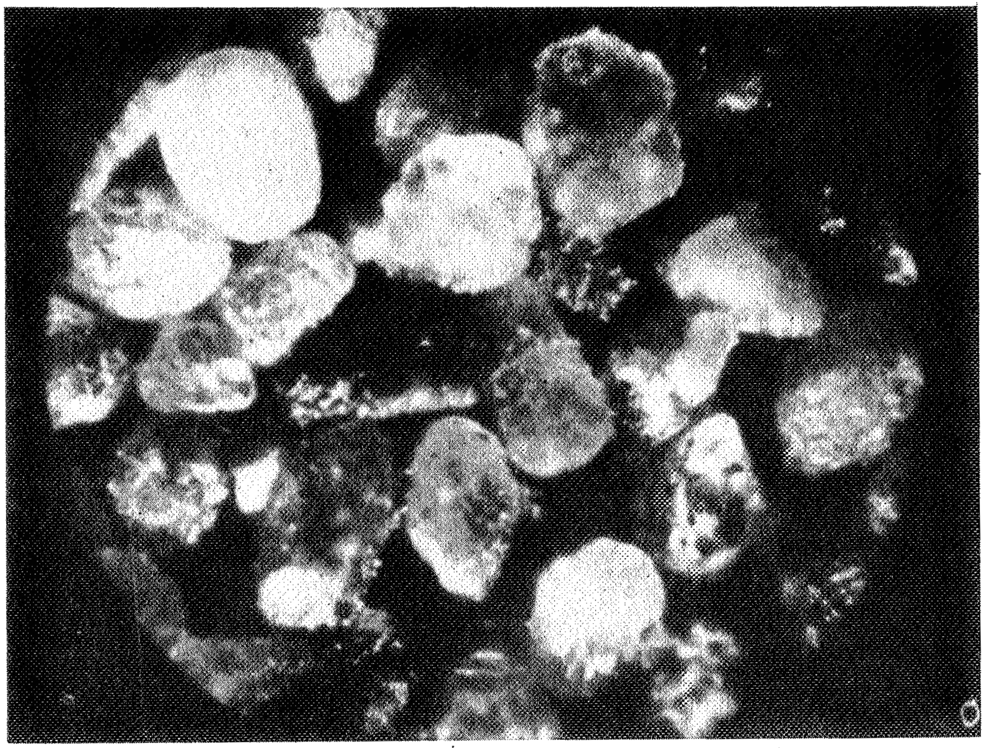

Figure 4.- Microphotograph of sand grains which produce uniform roughness. (Magnified about 20 times.)

7

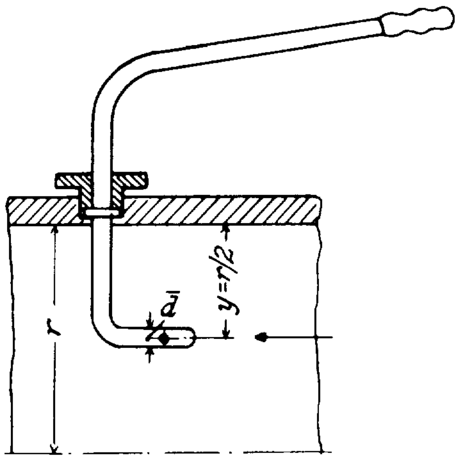

Figure 5.- Hooked tube for measuring static pressure (distance y between wall and observation point is $\frac{r}{2}$).

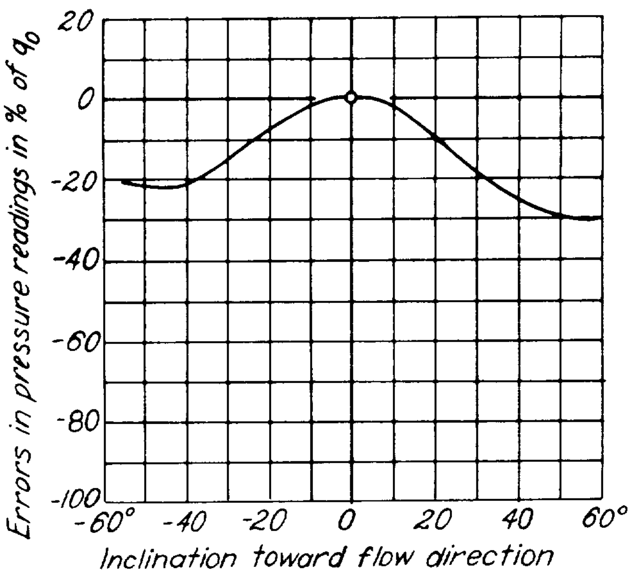

Figure 6.- Variation of readings with direction of hooked tube.

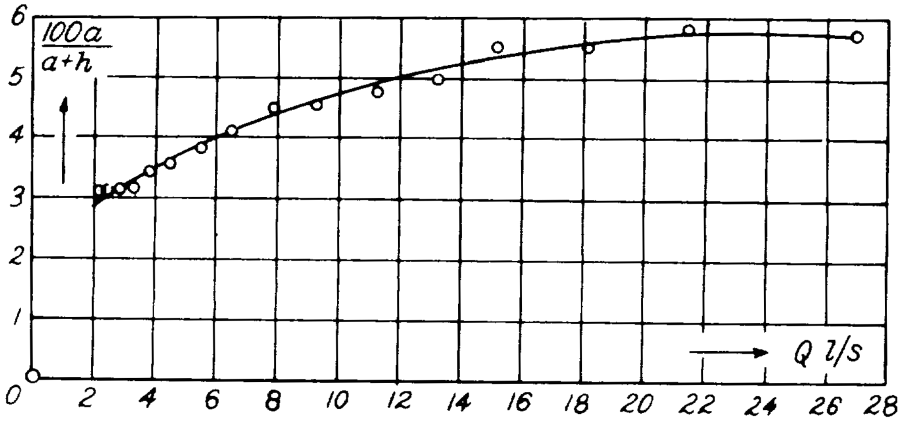

Figure 7.- Correction curve for determining static pressure.

a is resistance of hooked tube
h is resistance of smooth pipe

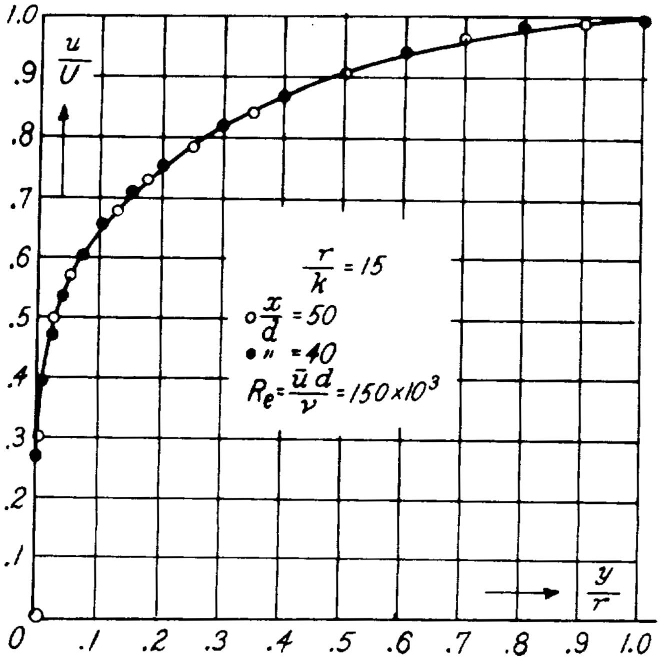

Figure 8.- Velocity distribution with $\frac{x}{d} = 40$ and $\frac{x}{d} = 50$ for $\frac{r}{k} = 15$ and $Re = 150 \times 10^3$ (y is distance between wall and observation point).

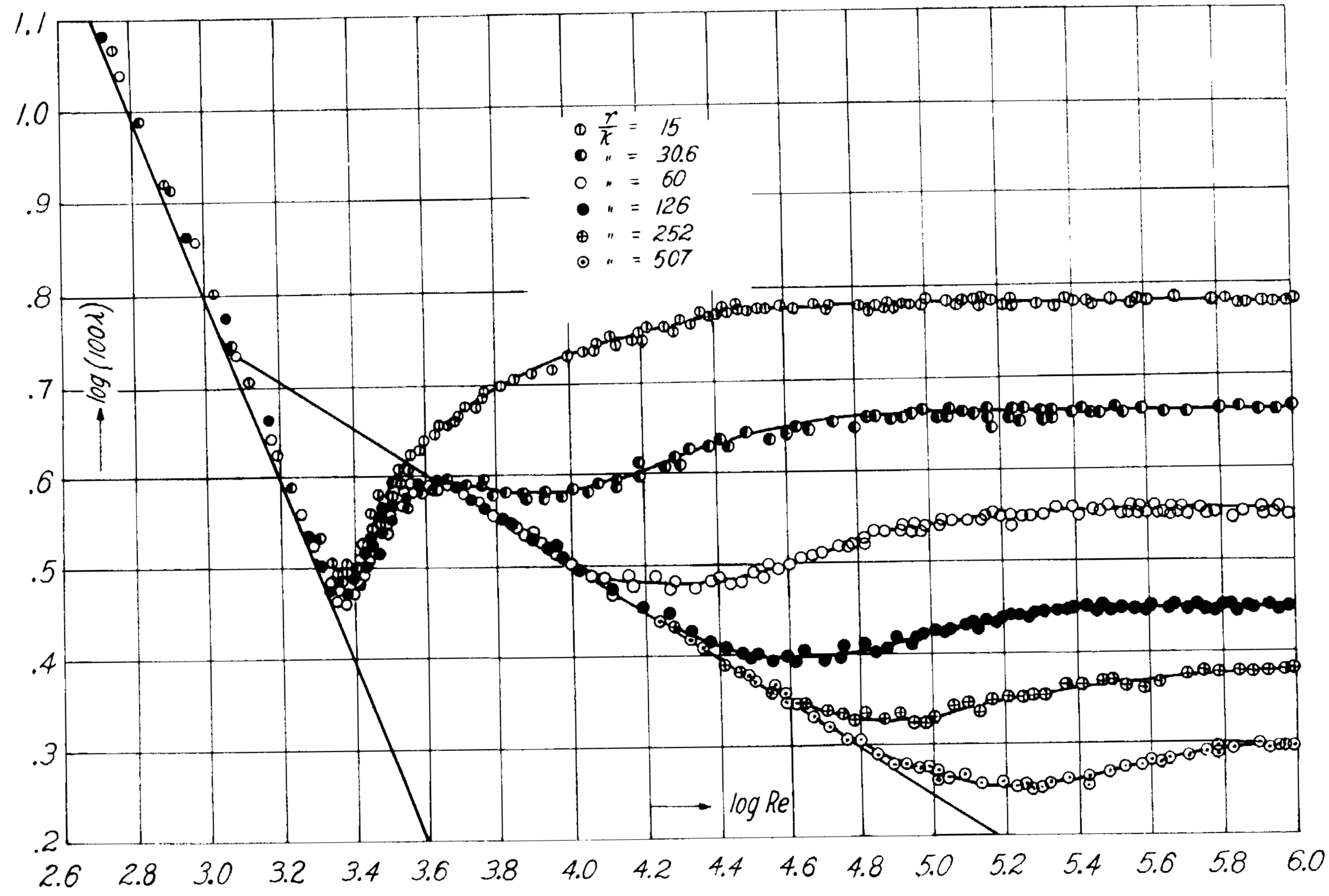

Figure 9.- Relation between log(100 λ) and log Re.

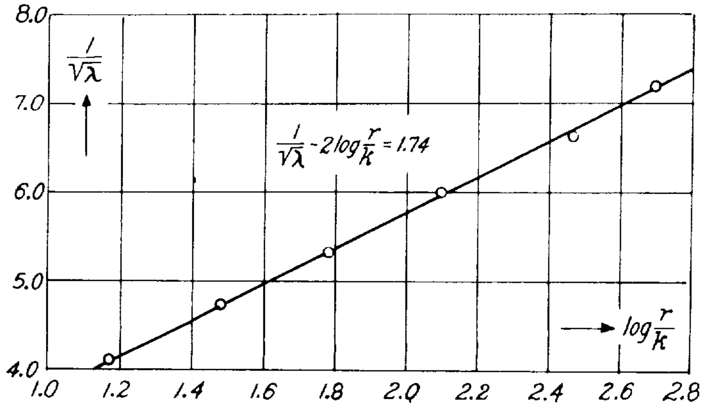

Figure 10.- Relation between $\frac{1}{\sqrt{\lambda}}$ and $\log \frac{r}{k}$.

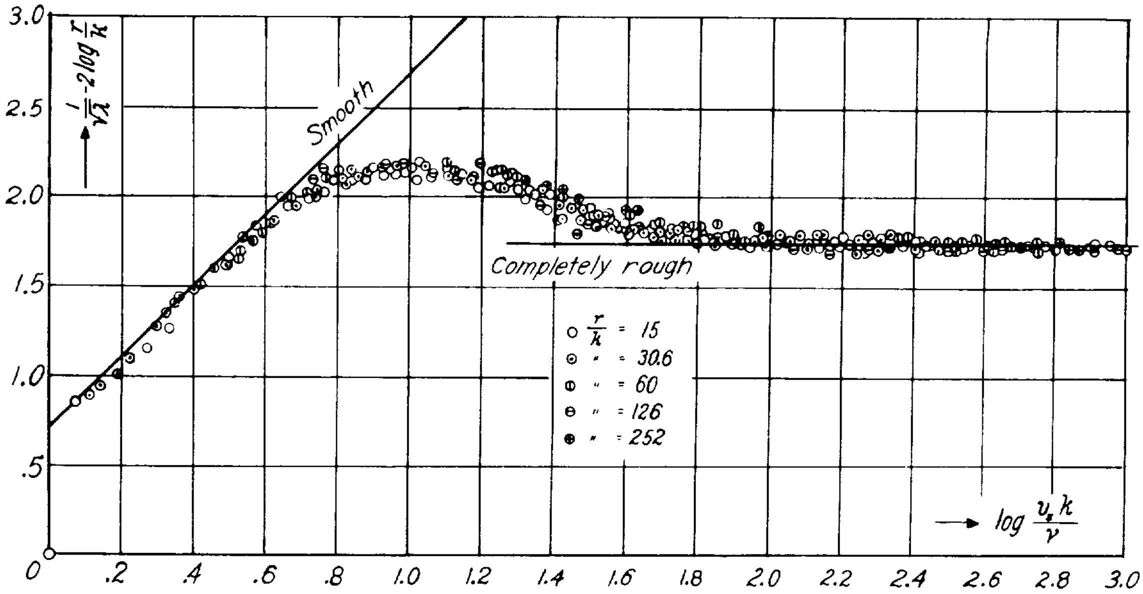

Figure 11.- Relation between $\left(\frac{1}{\sqrt{\lambda}} - 2 \log \frac{r}{k}\right)$ and $\left(\log \frac{v_* k}{\nu}\right)$.

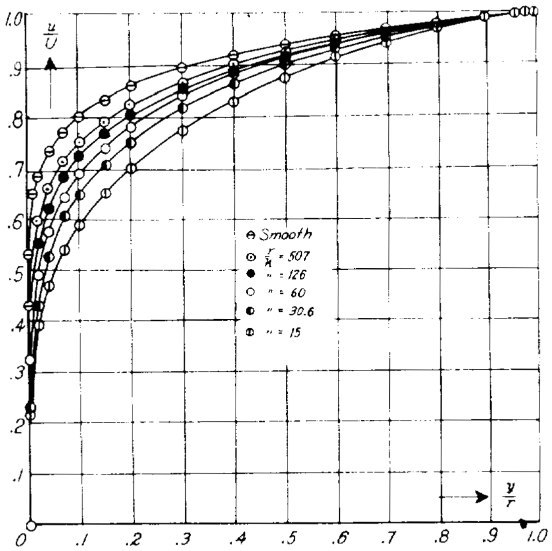

Figure 12.- Relation between $\frac{u}{U}$ and $\frac{y}{r}$ within the region of the quadratic law of resistance.

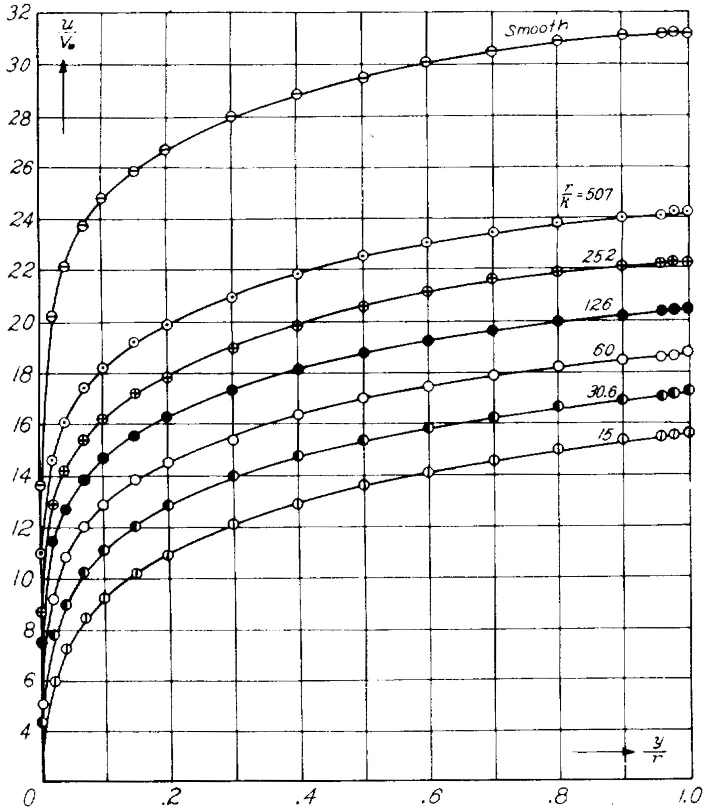

Figure 13.- Relation between $\frac{u}{v_*}$ and $\frac{y}{r}$.

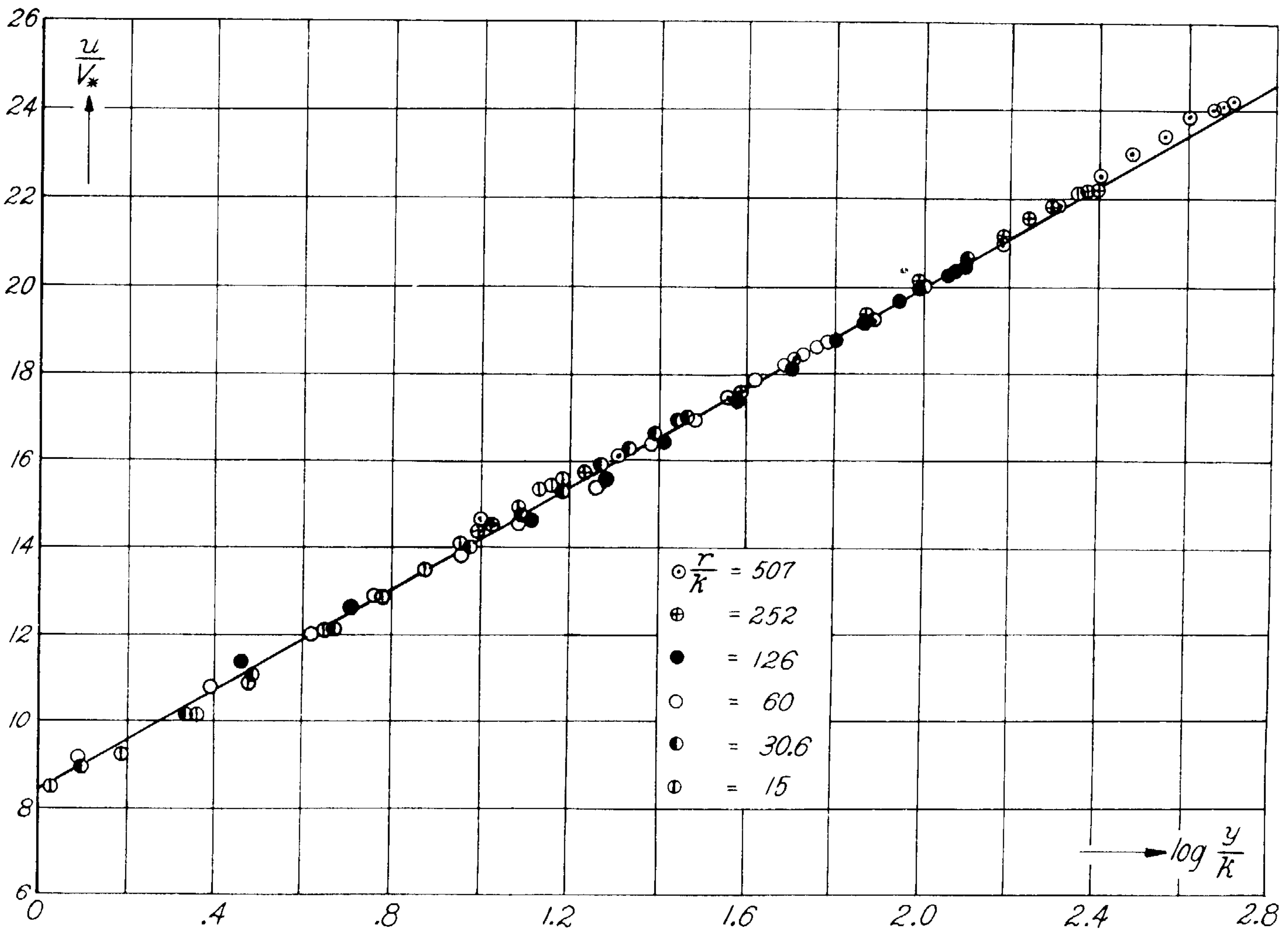

Figure 14.- Relation between $\frac{u}{V_*}$ and $\log \frac{y}{k}$.

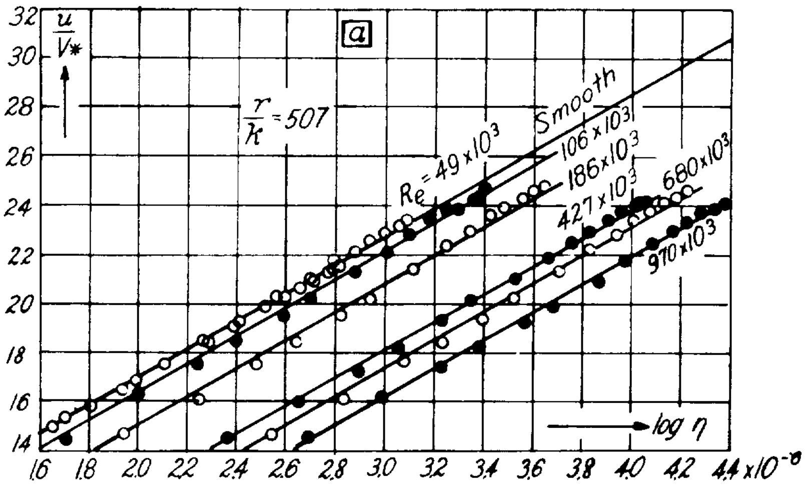

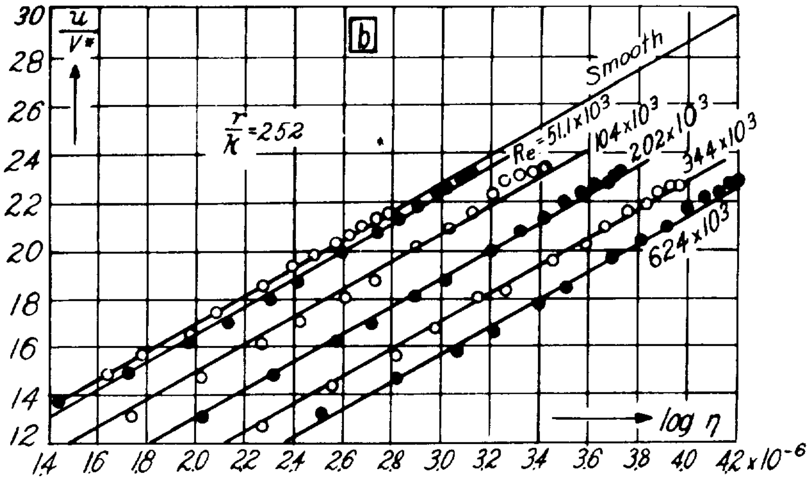

Figure 15.- Relation between $\frac{u}{v_*}$ and log η.

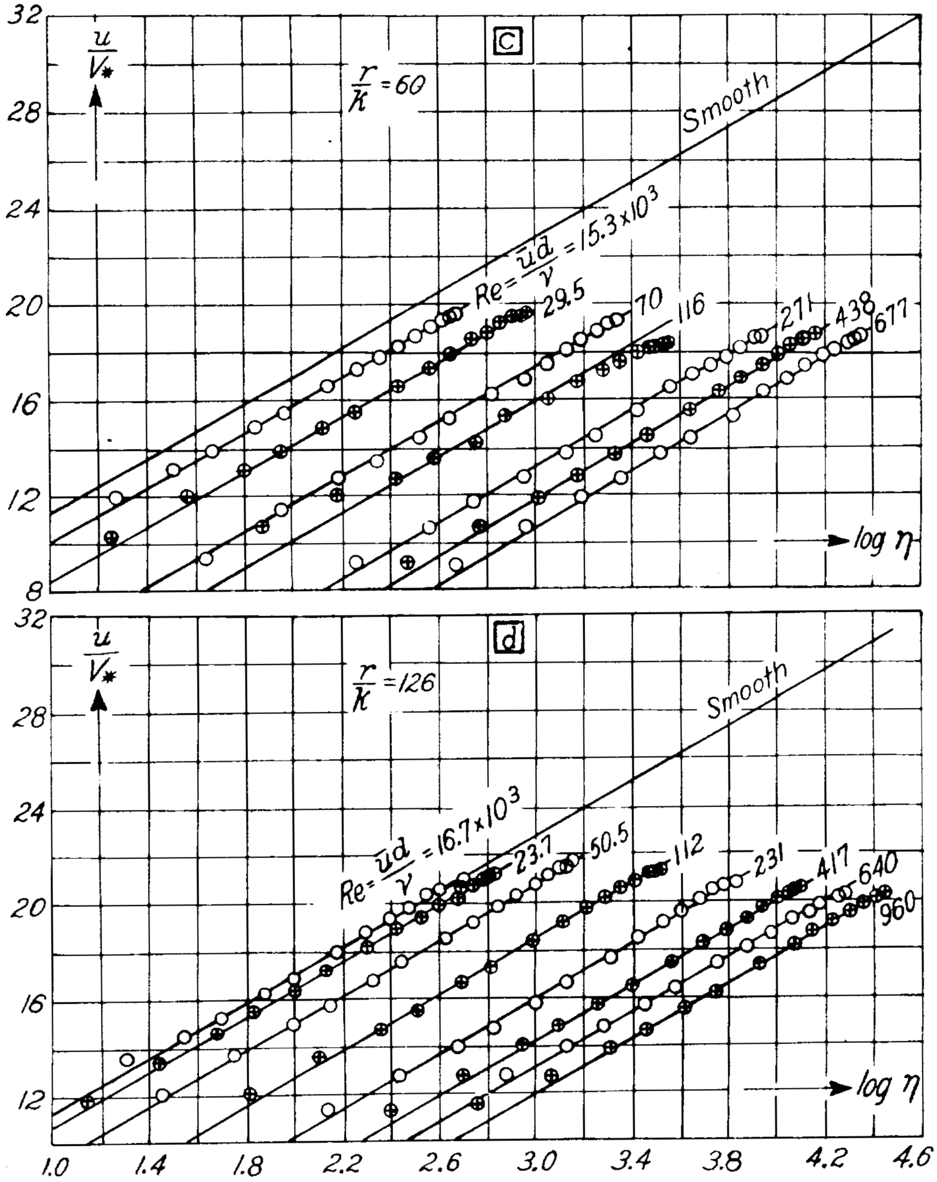

Figure 15.- Continued.

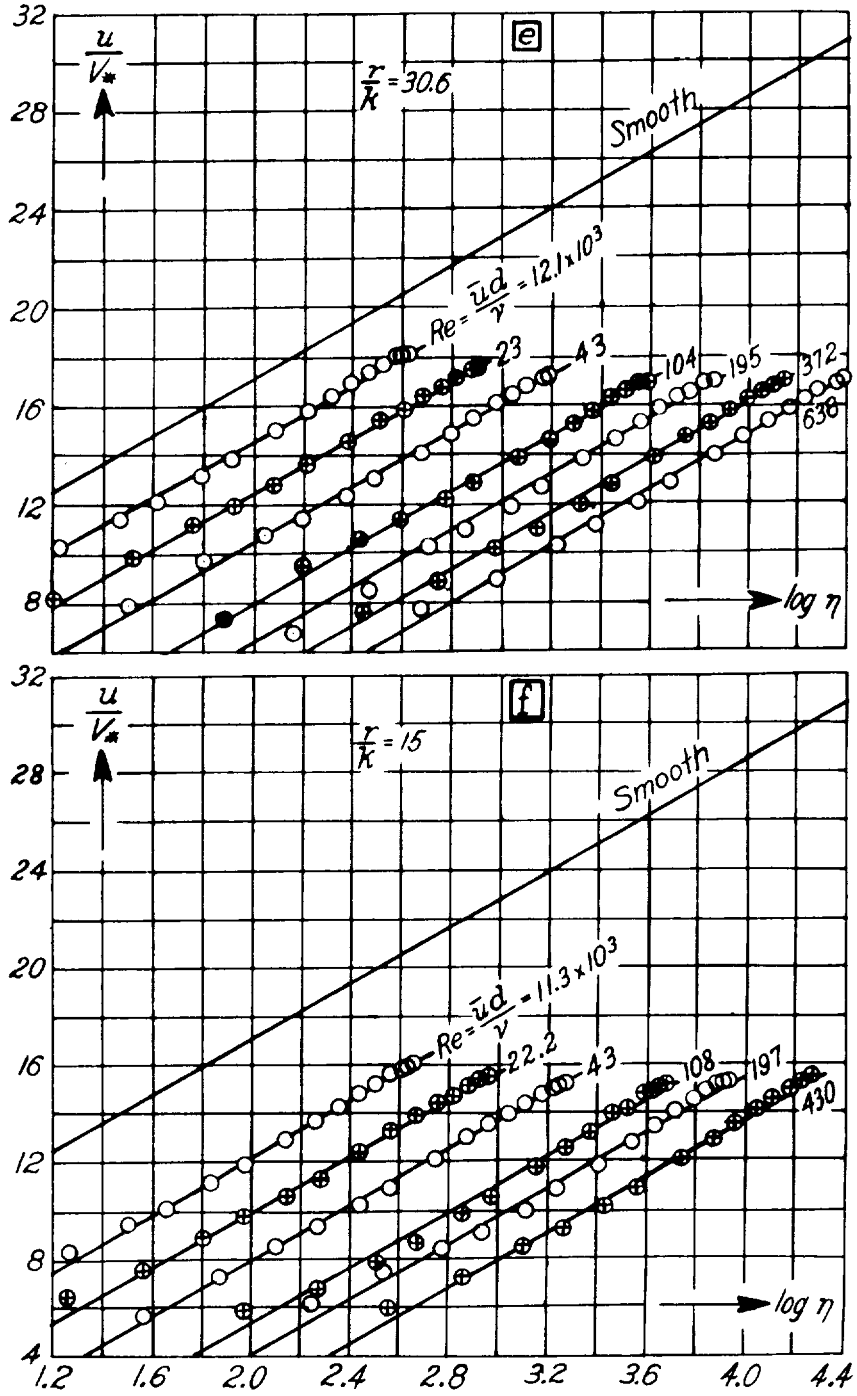

Figure 15.- Concluded.

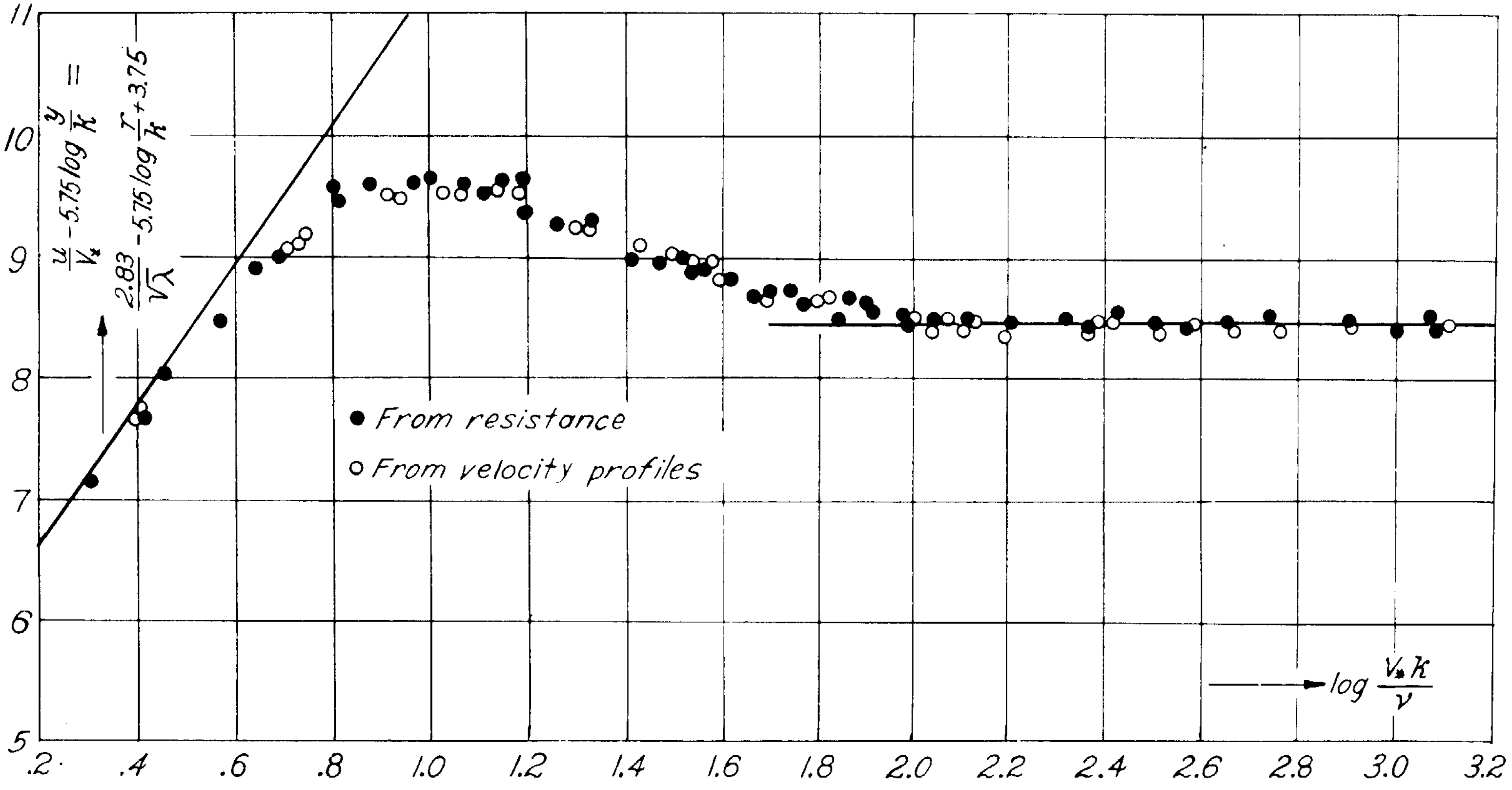

Figure 16.- Relation between $\left(\frac{u}{v_*} - 5.75 \log \frac{y}{k} = \frac{2.83}{\sqrt{\lambda}} - 5.75 \log \frac{r}{k} + 3.75\right)$ and $\left(\log \frac{v_* k}{\nu}\right)$.

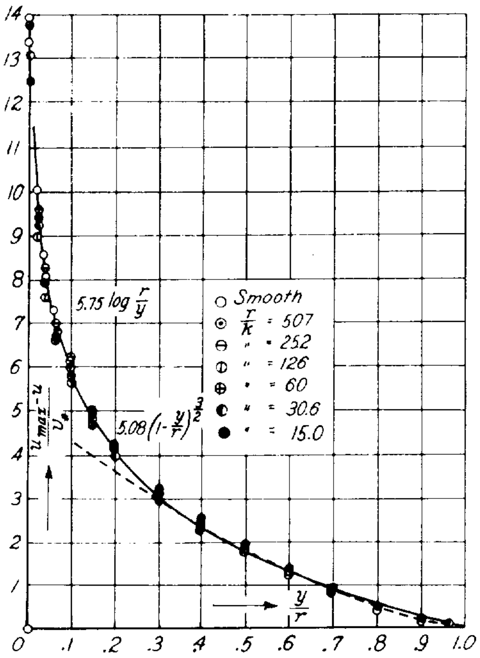

Figure 17.- Relation between $\left(\frac{u_{max} - u}{v_*} = \frac{U - u}{v_*}\right)$ and $\left(\frac{y}{r}\right)$.

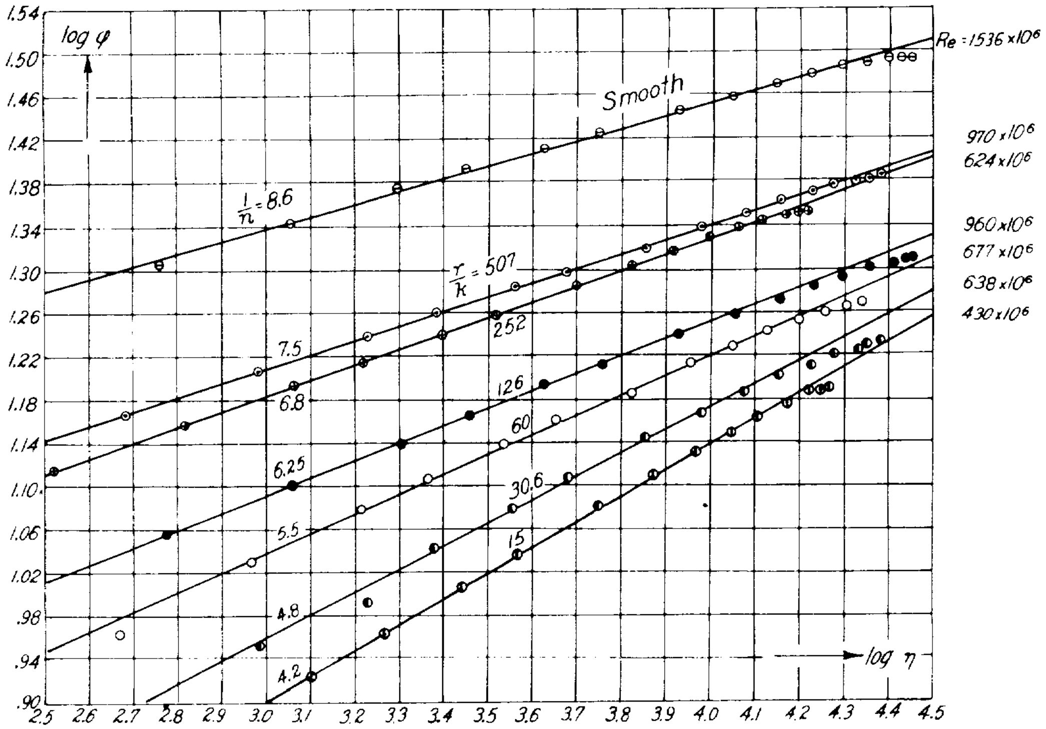

Figure 18.- Relation between log φ and log η for various degrees of roughness.

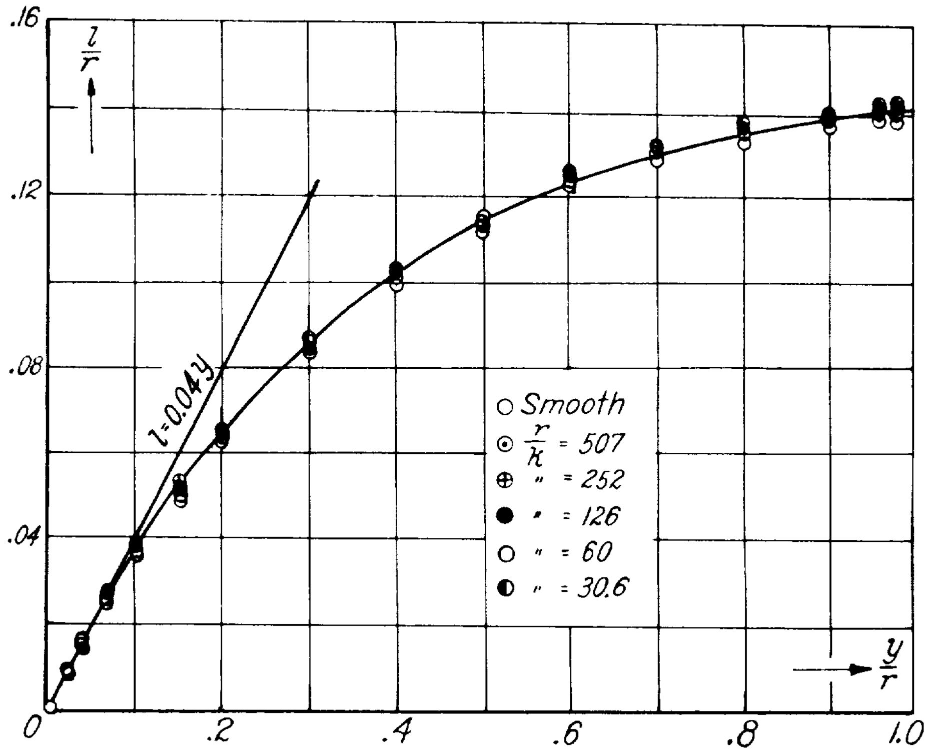

Figure 19.- Relation between $\frac{l}{r}$ and $\frac{y}{r}$ for large Reynolds numbers.

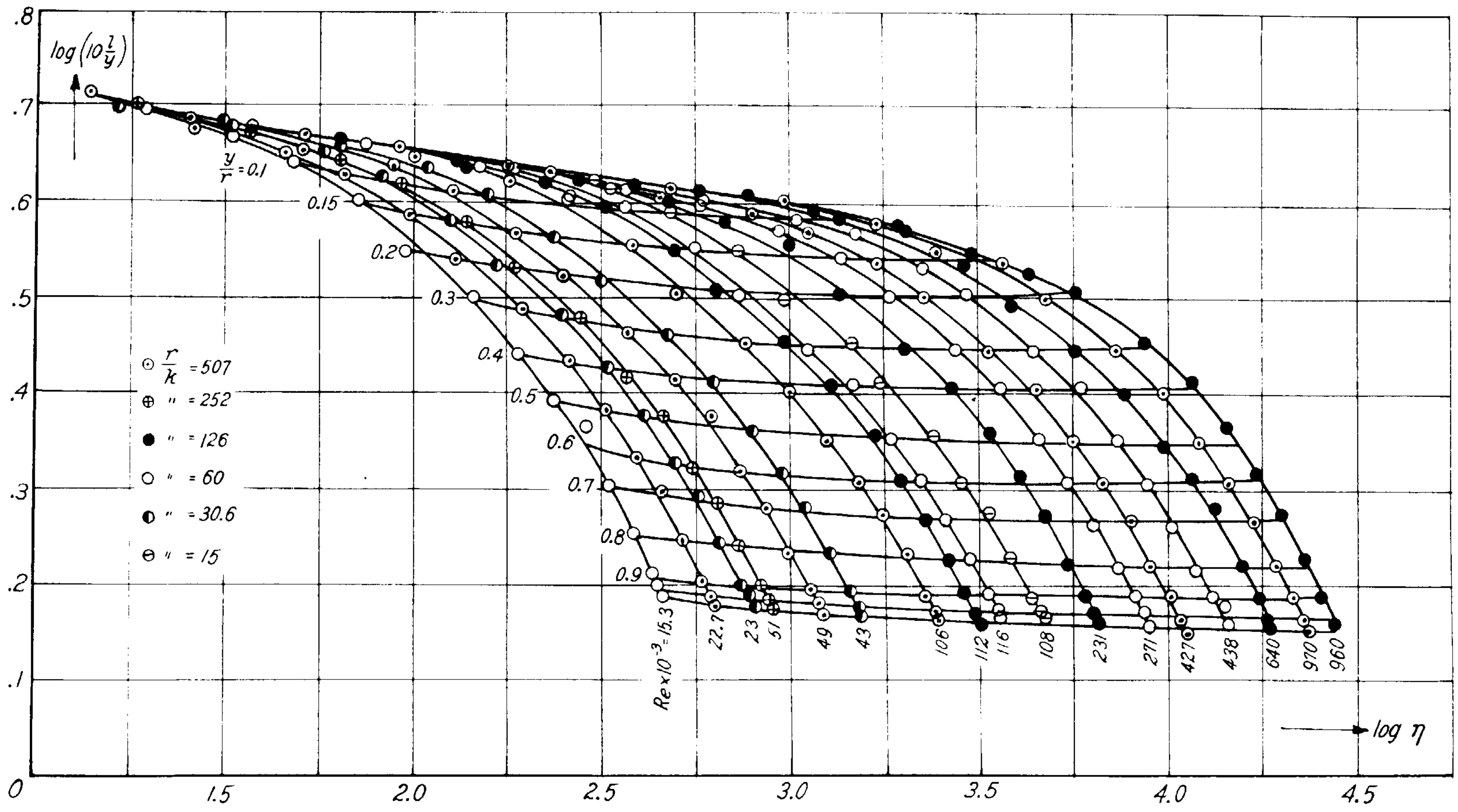

Figure 20.- Relation between $\log\left(10\frac{l}{y}\right)$ and $\log\eta$.

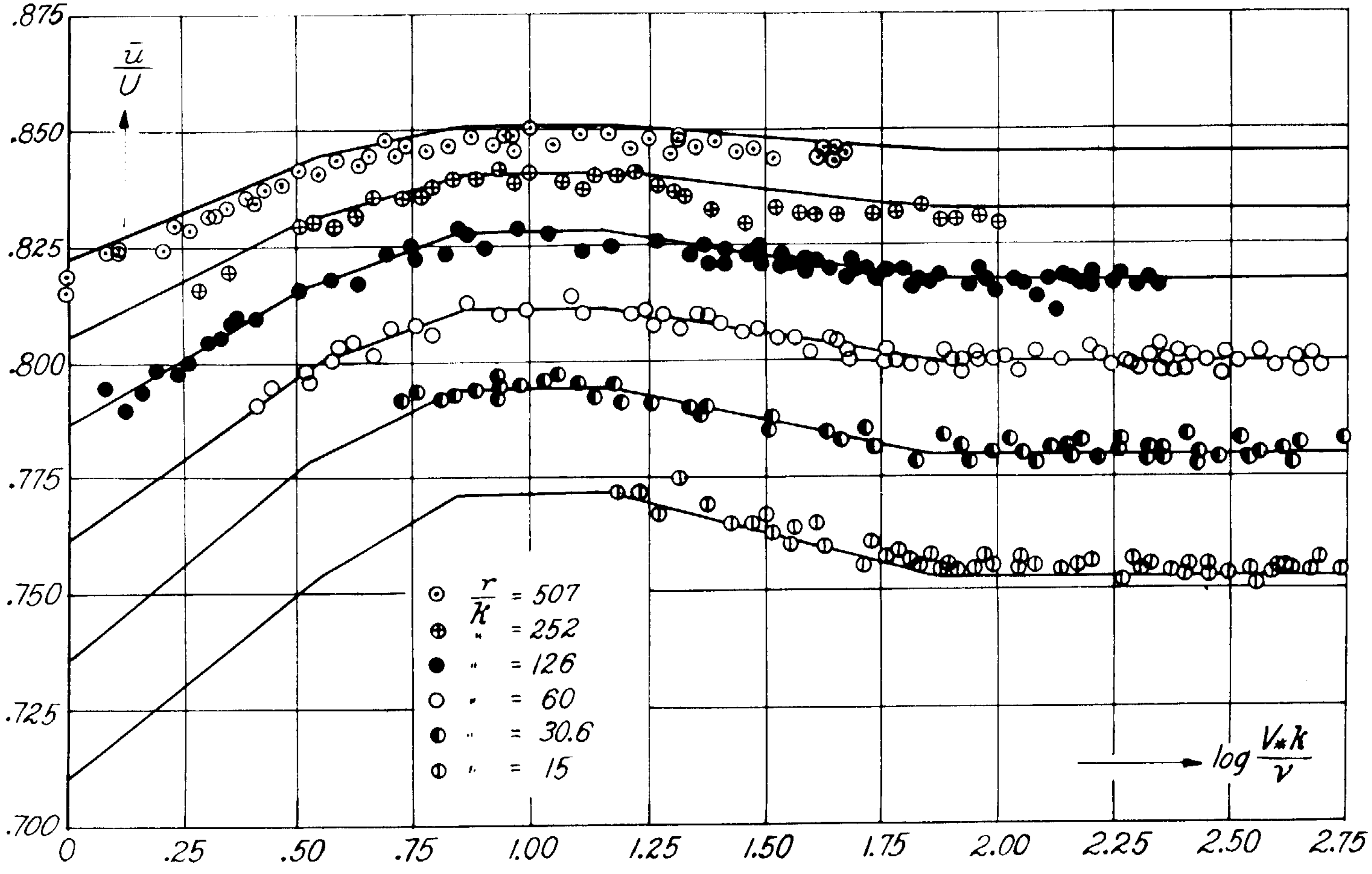

Figure 21.- Relation between $\frac{\bar{u}}{U}$ and $\log \frac{v_* k}{\nu}$.

NACA-Langley - 11-28-50 - 1000
